# Coordinated metabolic transitions and gene expression by NAD^+^ during adipogenesis

**DOI:** 10.1083/jcb.202111137

**Published:** 2022-10-05

**Authors:** Edgar Sánchez-Ramírez, Thi Phuong Lien Ung, Alejandro Alarcón del Carmen, Ximena del Toro-Ríos, Guadalupe R. Fajardo-Orduña, Lilia G. Noriega, Victor A. Cortés-Morales, Armando R. Tovar, Juan José Montesinos, Ricardo Orozco-Solís, Chiara Stringari, Lorena Aguilar-Arnal

**Affiliations:** 1 Departamento de Biología Celular y Fisiología, Instituto de Investigaciones Biomédicas, Universidad Nacional Autónoma de México, Mexico City, Mexico; 2 Laboratory for Optics and Biosciences, Ecole polytechnique, CNRS, INSERM, Institut Polytechnique de Paris, Palaiseau, France; 3 Departamento de Fisiología de la Nutrición, Instituto Nacional de Ciencias Médicas y Nutrición Salvador Zubirán, Mexico City, Mexico; 4 Mesenchymal Stem Cells Laboratory, Oncology Research Unit, Oncology Hospital, National Medical Center, Mexico City, Mexico; 5 Laboratorio de Cronobiología y Metabolismo, Instituto Nacional de Medicina Genómica, Mexico City, Mexico

## Abstract

Adipocytes are the main cell type in adipose tissue, which is a critical regulator of metabolism, highly specialized in storing energy as fat. Adipocytes differentiate from multipotent mesenchymal stromal cells (hMSCs) through adipogenesis, a tightly controlled differentiation process involving close interplay between metabolic transitions and sequential programs of gene expression. However, the specific gears driving this interplay remain largely obscure. Additionally, the metabolite nicotinamide adenine dinucleotide (NAD^+^) is becoming increasingly recognized as a regulator of lipid metabolism, and a promising therapeutic target for dyslipidemia and obesity. Here, we explored how NAD^+^ bioavailability controls adipogenic differentiation from hMSC. We found a previously unappreciated repressive role for NAD^+^ on adipocyte commitment, while a functional NAD^+^-dependent deacetylase SIRT1 appeared crucial for terminal differentiation of pre-adipocytes. Repressing NAD^+^ biosynthesis during adipogenesis promoted the adipogenic transcriptional program, while two-photon microscopy and extracellular flux analyses suggest that SIRT1 activity mostly relies on the metabolic switch. Interestingly, SIRT1 controls subcellular compartmentalization of redox metabolism during adipogenesis.

## Teaser

Sánchez-Ramirez, Ung et al. show that NAD^+^ bioavailability controls adipogenic differentiation obstructing adipocyte commitment, downregulating ribosomal proteins–coding genes and leading to quiescent metabolic state. Instead, the NAD^+^-dependent SIRT1 deacetylase is essential for terminal differentiation of pre-adipocytes and regulates subcellular compartmentalization of redox metabolism during adipogenesis.

## Introduction

Adipose tissue is a crucial regulator of body metabolism through storing calories as lipids in response to excessive nutritional intake and serving as a source of energy by mobilizing these lipids during starvation, amongst others. Notably, the adipose tissue is a relevant endocrine organ, producing several adipokines such as leptin or adiponectin (Galic et al., 2010). It is primarily composed of adipocytes, and a fraction of a heterogeneous collection of cell types which include mesenchymal stem cells (MSCs), endothelial precursors, immune cells, smooth muscle cells, pericytes, and preadipocytes (Nguyen et al., 2016). Dysfunction of the adipose compartment is common to metabolic diseases including obesity or type 2 diabetes. Indeed, increased white adipose tissue mass observed in obesity is due to both adipocyte hypertrophy and increased proliferation and differentiation of adipocyte progenitors (Berry and Rodeheffer, 2013; Rodeheffer et al., 2008), which originate from MSCs through the adipogenic process (Cristancho and Lazar, 2011; Peirce et al., 2014). Hence, understanding the mechanisms underlying adipogenesis in humans is crucial to design therapeutic strategies for prevalent metabolic dysfunctions.

MSCs are multipotent progenitor cells able to differentiate into osteoblasts, myocytes, chondrocytes, and adipocytes. Fate decision is determined by specific signaling pathways such as TGFβ/bone morphogenic protein (BMP) signaling, wingless-type mouse mammary tumor virus integration site (Wnt) signaling or FGFs (Chen et al., 2016; Ghaben and Scherer, 2019). In particular, the adipogenic process occurs in two major phases: commitment to progenitors and terminal differentiation, both of which are tightly regulated by intertwined transcriptional, epigenomic, and metabolic transitions (Ghaben and Scherer, 2019). At the transcriptional level, the master regulators of adipogenesis are the key transcription factors peroxisome proliferator-activated receptor γ (PPARγ) and CCAAT/enhancer binding protein α (C/EBPα; Morrison and Farmer, 1999; Tontonoz et al., 1994), which promote growth arrest and the progressive expression of a lipogenic transcriptional program including the hormones adiponectin and leptin, and the lipases adipose triglyceride lipase and lipoprotein lipase (LPL; Ghaben and Scherer, 2019). Concomitantly, a switch from highly glycolytic to oxidative metabolism with increased mitochondrial reactive oxygen species (ROS) is essential for adipocyte differentiation (Shyh-Chang et al., 2013; Tormos et al., 2011). However, how transcriptional and metabolic transitions reciprocally interact during adipogenic differentiation remains an open question.

During the past few years, energy metabolism is becoming increasingly recognized as an effective therapeutic target for obesity. Specifically, therapies aiming to increase endogenous nicotinamide adenine dinucleotide (NAD^+^) levels have been proven effective to reduce adiposity in both mouse and human (Cantó et al., 2012; Gariani et al., 2016; Remie et al., 2020; Trammell et al., 2016). Indeed, NAD^+^ levels decline in metabolic tissues of obese mice and humans (Gariani et al., 2016; Gulshan et al., 2018; Jukarainen et al., 2016; Liu et al., 2017; Trammell et al., 2016; Yoshino et al., 2011), which may contribute to metabolic dysfunction by, for example, reducing the activity of SIRT1, a deacetylase using NAD^+^ as a substrate and known to regulate mitochondrial function and metabolism (Chang and Guarente, 2014; Giblin et al., 2014). This evidence suggests that NAD^+^ metabolism might be a central player on adipose tissue homeostasis probably by regulating mitochondrial function and consequently, adipocyte differentiation. Along these lines, in mouse preadipocytes, NAD^+^ synthesis through the salvage pathway and SIRT1 activity appear essential for adipogenesis (Okabe et al., 2020); however, the interplay between NAD^+^ bioavailability and SIRT1 function during adipogenesis in humans remains poorly understood.

In this study, we explored the coordinated dynamics between transcriptional and energy metabolism reprogramming during adipogenic differentiation of human MSC (hMSC). Using two-photon fluorescence lifetime microscopy (2P-FLIM; Skala et al., 2007; Stringari et al., 2011; Stringari et al., 2012) on live hMSC, we created a non-invasive metabolic map of NADH compartmentalization at a submicron resolution to define the dynamics of redox metabolism during adipogenesis, which appeared tightly synchronized with mitochondrial function and transcriptional reprogramming. Moreover, we described a previously unappreciated robust inhibitory role for NAD^+^ on adipocyte commitment, while SIRT1 activity appeared essential for terminal differentiation. Surprisingly, suppressing the NAD^+^ salvage pathway during adipogenesis led to increased expression of adipocyte markers, indicating that SIRT1 activation during adipogenesis does not depend on NAD^+^ biosynthesis through the salvage pathway. Remarkably, SIRT1-directed control of compartmentalization of redox metabolism during adipogenesis was evidenced by 2P-FLIM.

## Results

### NAD^+^ obstructs adipogenic differentiation and lipid accumulation in hMSC

**Figure 1.**
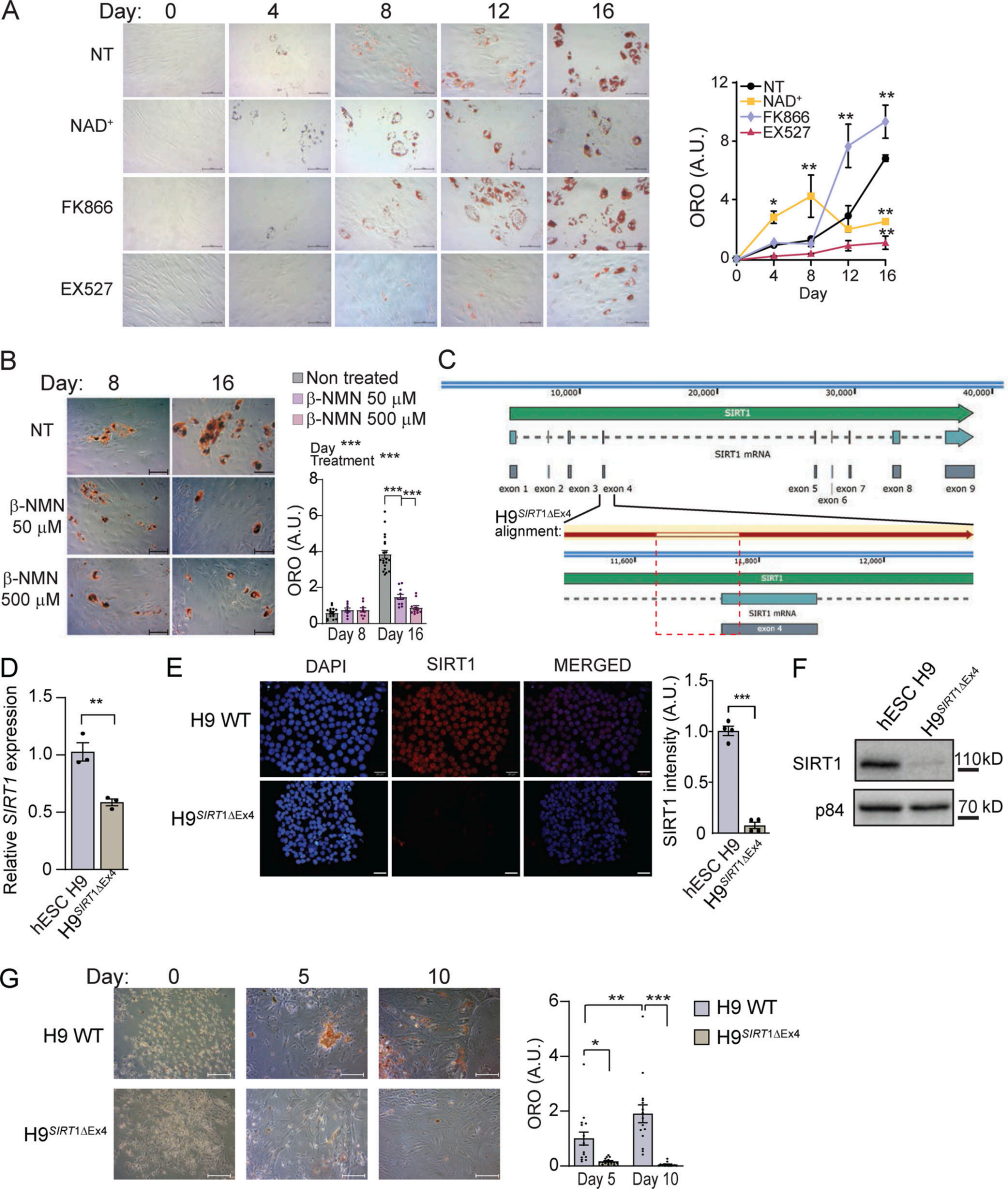
**NAD**^**+**^**-SIRT1 pathway shapes adipogenic differentiation and lipid accumulation in hMSC.** Adipogenic differentiation was induced in hMSC in the absence or presence of the indicated drugs: NAD^+^ (5 mM), FK866 (1 nM), the SIRT1-specific inhibitor EX527 (50 μM), or β-NMN (50 or 500 μM), as indicated. **(A and B)** Neutral lipids were stained with ORO at the indicated days after adipogenic induction, and representative images are shown. Quantification was performed by densitometry from *n* = 4 technical and 3 biological replicates. Scale bars are for 50 μm. **(C)** Targeted mutation of the exon 4 in SIRT1 gene using a CRISPR-Cas9 strategy in H9 hESCs. The structure of the locus is represented together with the alignment of the sequence in the H9^*SIRT1*ΔEx4^ (red arrow), and the deleted is indicated within the red dashed rectangle. **(D)** *SIRT1* mRNA expression in H9 WT and H9^*SIRT1*ΔEx4^ cells measured by qRT-PCR. Data were normalized to *Tbp* expression, and H9 data were set to 1. **(E and F)** SIRT1 protein expression in H9 WT and H9^*SIRT1*ΔEx4^ cells was analyzed by immunofluorescence with scale bars for 30 μm (E) or Western blot (F). **(G)** Neutral lipids were stained with ORO at the indicated days after adipogenic induction in hESC, and representative images are shown. Quantification was performed by densitometry from *n* = 10 technical and 3 biological replicates. Scale bars are for 50 μm. For all graphs, data are presented as mean ± SEM (*P < 0.05, **P < 0.01, ***P < 0.001, two-way ANOVA with Tukey’s or Bonferroni’s post-test). Source data are available for this figure: SourceData F1.

To approach the question of whether variations in NAD^+^ bioavailability impact adipogenesis, we induced adipogenic differentiation in hMSCs in the presence or absence of NAD^+^ (Fig. 1 A). Interestingly, NAD^+^ treatment obstructed neutral lipid accumulation, as visualized by significantly less accumulation of Oil-red-O (ORO) stain at terminal differentiation than in non-treated (NT) cells (Fig. 1 A; day 16, P < 0.01, two-way ANOVA with Tukey’s post-test). Concomitantly, treatment with FK866, a potent and highly selective inhibitor of NAMPT (Aguilar-Arnal et al., 2016; Hasmann and Schemainda, 2003), the rate-limiting enzyme in the NAD^+^ salvage biosynthetic pathway, led to significantly more neutral lipids at day 16 (Fig. 1 A; day 16, P < 0.01, two-way ANOVA with Tukey’s post-test), further reinforcing the idea that NAD^+^ bioavailability might oppose lipogenesis. As it has been largely shown that SIRT1 is a major effector of NAD^+^ signaling, we reasoned that it might be dispensable for adipogenesis. Surprisingly, SIRT1 inhibition by EX527, a potent and selective small molecule inhibiting SIRT1 activity (Napper et al., 2005), during adipogenic differentiation strongly hindered neutral lipid accumulation (Fig. 1 A; day 16, P < 0.01, two-way ANOVA with Tukey’s post-test). Interestingly, distinct dynamic changes in lipid accumulation were observed at days 4, 8, and 12 of adipogenic inductions with the different treatments (Fig. 1 A). A treatment with β-nicotinamide mononucleotide (β-NMN), a NAD^+^ precursor with potential as a treatment for obesity-associated pathologies (Yoshino et al., 2011; Yoshino et al., 2021), also opposed adipogenesis in hMSCs (Fig. 1 B).

While treatments with EX527 have been widely used to specifically inhibit SIRT1, the precise pharmacological properties of this inhibitor remain unclear. To directly corroborate that SIRT1 is involved in adipogenic differentiation, we used a CRISPR-Cas9 approach to eliminate SIRT1 in the H9 human embryonic stem cell line (hESC; see Materials and methods section). The strategy deleted 133 bp at the junction between intron 3 and exon 4 of *SIRT1* gene, eliminating the amino acids 264–272 within the SIRT1 NAD^+^-binding domain (Fig. 1 C). This resulted in a 50% decrease of *SIRT1* gene expression (Fig. 1 D) while the SIRT1 protein was non-detectable in these *SIRT1* knockdown cells (H9^*SIRT1*ΔEx4^; Fig. 1, E and F). Similarly to what was observed in the EX527-treated hMSC, ORO staining at two sequential stages after adipogenic differentiation in H9WT and H9^*SIRT1*ΔEx4^ hESCs indicated that SIRT1 is essential to develop mature adipocytes (Fig. 1 G).

**Figure 2.**
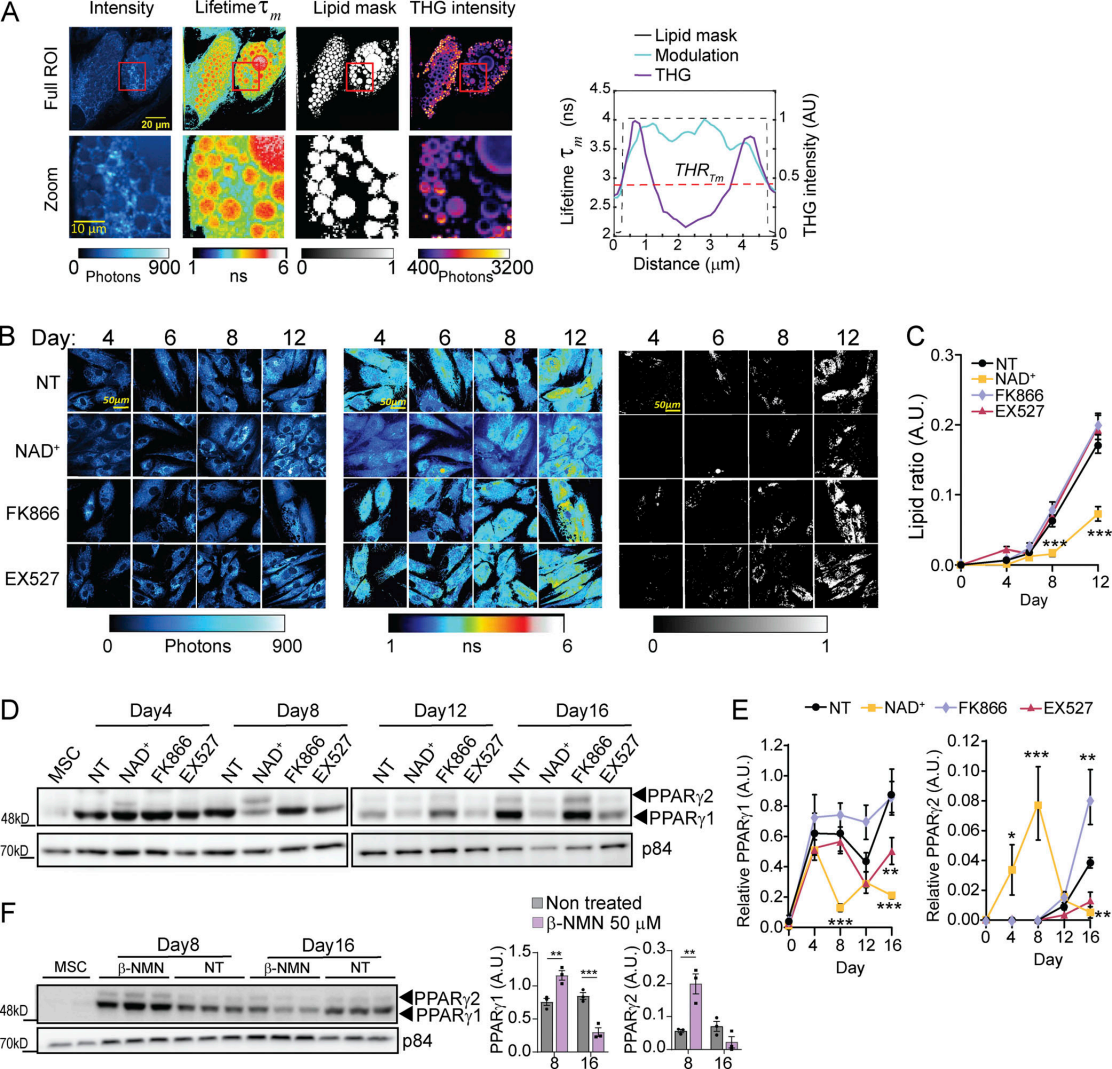
**NAD**^**+**^ **hinders lipid accumulation and PPARγ expression during adipogenesis in hMSCs. (A)** Representative images from label-free quantification of lipid droplets by FLIM of an adipocyte at terminal differentiation. Intensity, modulation lifetime tauM (τ_m_) lipid mask determined by a τ_m_ threshold, and THG intensity are shown as indicated. The graph on the right shows a cross-sectional profile of lipid mask created by a τ_m_ threshold and THG signal of one lipid droplet of 5 µm diameter. **(B)** Representative images of intensity (left), modulation lifetime t_m_ (middle), and lipid mask (right) of hMSCs during adipogenic differentiation at the indicated days of culture under selected treatments. **(C)** Quantification from lipid ratio at indicated days and treatments during the differentiation process of hMSC. *n* = 14 single cells; experiments were conducted in triplicate. **(D and F)** PPARγ1 and PPARγ2 protein expression levels were measured by Western blot in whole-cell extracts at the indicated days after adipogenic induction. p84 was used as loading control. **(E)** Quantification of PPARγ1 and PPARγ2 protein expression, normalized to p84 loading control. *n* = 3 biological replicates. For all graphs, data are presented as mean ± SEM (*P < 0.05, **P < 0.01, ***P < 0.001, two-way ANOVA with Tukey’s post-test). Source data are available for this figure: SourceData F2.

**Figure S1.**
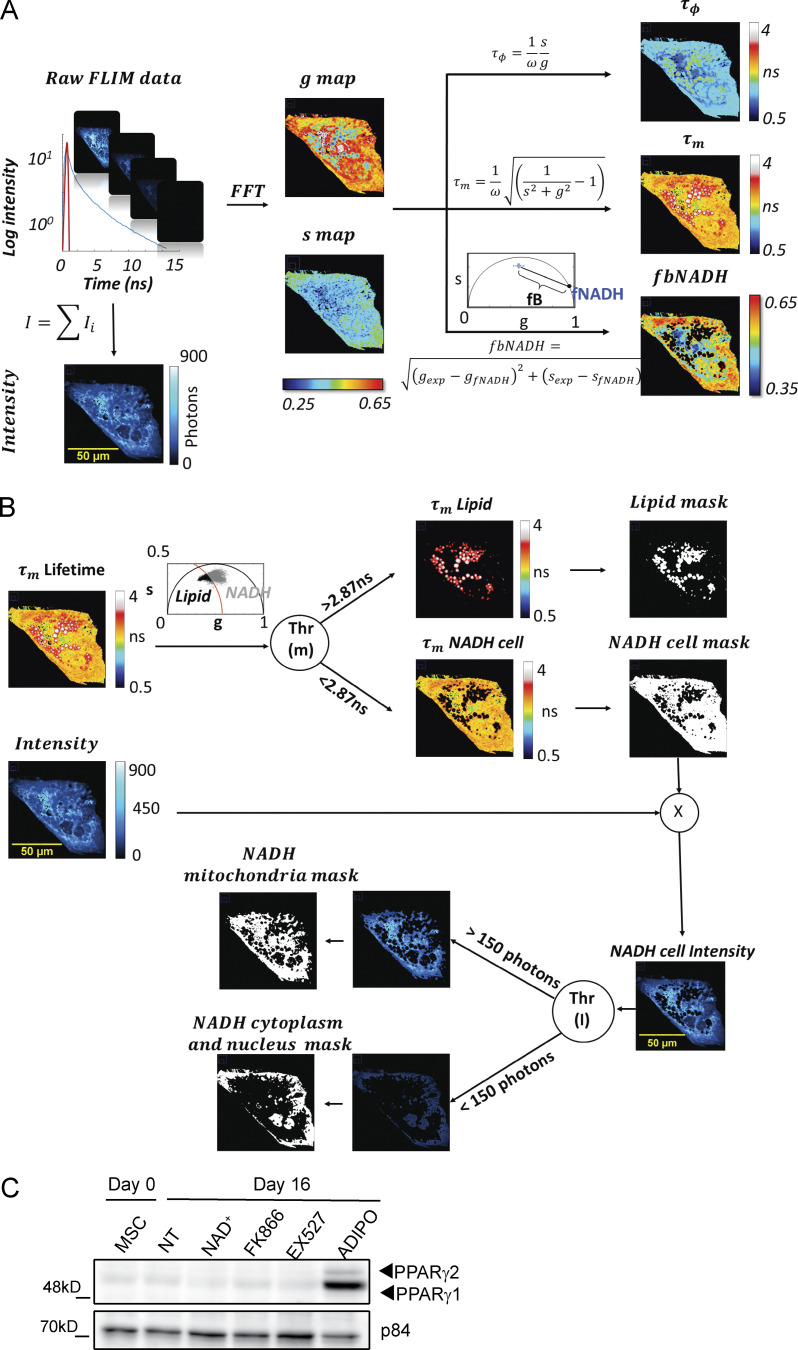
**Image processing workflow for FLIM and Lipid and NADH segmentation. (A)** Workflow of FFT-based Phasor analysis of FLIM images. **(B)** Workflow of subcellular segmentation based on lifetime and intensity thresholds. A threshold on modulation lifetime (THR [m] = 2.87 ns) is applied to separate lipid droplets and NADH in entire cell. The pixels of the FLIM image with τ_m_ > THR (m) (black points in the Phasor plot) are assigned to lipid droplets while the pixels with τ_m_ < THR (m) (gray points in the Phasor plot) are assigned to the NADH signal in the rest of the cell. A threshold (150 photons) on intensity of cell NADH is applied to segment mitochondria and nucleus plus cytoplasm. The pixels with intensity > THR (I) are assigned to mitochondria while pixels with intensity < THR (I) are assigned to the cytoplasm and nucleus. **(C)** PPARγ1 and PPARγ2 protein expression levels were measured by Western blot in whole cell extracts from hMSC untreated (NT) or treated with the indicated compounds for 16 d. Terminally differentiated adipocytes (ADIPO) were also included. p84 was used as loading control. Source data are available for this figure: SourceData FS1.

To further confirm that lipid accumulation is changing with the treatments in living cells while avoiding potential artifacts from fixation or ORO stain, we implemented label-free FLIM of intrinsic lipid-associated fluorophores in living cells (Alonzo et al., 2016; Datta et al., 2015; see Materials and methods section). Third harmonic generation (THG) microscopy images were used as reference to identify lipid droplets (Chang et al., 2013; Débarre et al., 2006; Fig. 2 A; and Fig. S1, A and B). The THG contrast present bright round dots for lipid droplets smaller than 1 µm and hollow round structures at the interface between cytoplasm and lipid droplets larger than 1 µm. FLIM images (Fig. 2 A) indicated that lipid droplets associated fluorophores have a longer lifetime (τ_m_) with respect to the rest of the cell cytoplasm; hence, we determined a threshold to automatically and systematically identify and segment lipid droplets (Fig. 2 A and Fig. S1 B, Materials and methods section). This method allowed us to quantitively assess lipid accumulation in live hMSCs during adipogenic induction (Fig. 2 B). We imagined cells at days 4, 6, 8, and 12, corresponding to those when more dynamic changes were previously observed, and found that NAD^+^ treatment significantly impaired lipid accumulation after day 8 (Fig. 2, B and C; day 8 and 12, P < 0.001, two-way ANOVA with Tukey’s post-test). With this approach, we did not find significant differences in lipid accumulation in live cells when comparing FK866 and EX527 treatments to NT cells, although these lipids could still be distinct in droplet size or composition.

PPARγ is considered a master transcription factor for adipogenesis (Lefterova et al., 2014; Lefterova et al., 2008). Its expression is induced in early stages (Aprile et al., 2014) and sustains the specific transcriptional program for adipocyte differentiation (Tontonoz and Spiegelman, 2008). For these reasons, we explored how NAD^+^ levels impact PPARγ protein expression during adipogenic induction of hMSCs (Fig. 2, D and E). We found that PPARγ1 isoform is strongly expressed since day 4 of differentiation for all tested conditions (Fig. 2, D and E). Yet, while its expression was overall sustained along the adipogenic process in control cells, NAD^+^ triggers a very significant reduction in PPARγ1 protein expression while favoring expression of the PPARγ2 isoform at differentiation day 8, which is lost at later stages (Fig. 2, D and E; P < 0.01, two-way ANOVA with Tukey’s post-test). Similar to NAD^+^, β-NMN treatment also promoted PPARγ2 isoform expression at day 8, while PPARγ1 was significantly decreased at the end of the adipogenic differentiation (Fig. 2 F).Interestingly, EX527 treatment led to significantly decreased expression of PPARγ1 at the end of differentiation. Conversely, FK866 significantly increased PPARγ2 isoform with respect to the control without affecting PPARγ1 protein expression. As expected, a 16-d treatment of hMSC with the compounds led to no detectable changes in PPARγ expression (Fig. S1 C). These differential dynamics on PPARγ protein expression point to a major role for transcriptional control in the loss of adipogenic potential after NAD^+^ treatment, which is also in line with the distinct outcomes in lipid accumulation.

### Extensive and specific reprogramming of the transcriptome during adipogenic differentiation by NAD^+^

**Figure 3.**
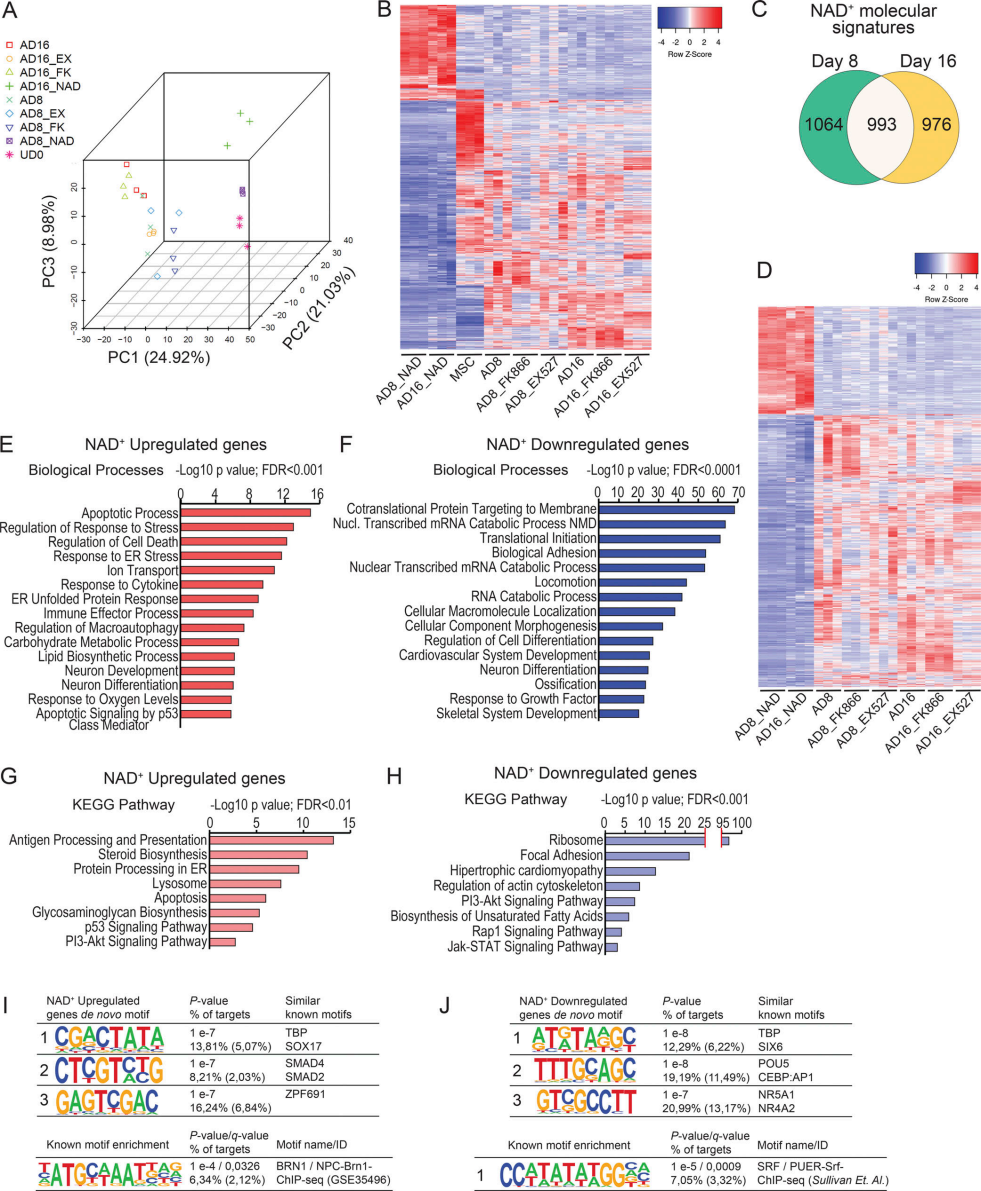
**NAD**^**+**^ **treatment during adipogenesis from hMSC induces profound and specific changes in the transcriptome. (A)** RNA-seq was performed per triplicate from multipotent hMSC (MSC, UD0), or at days 8 (AD8) and 16 (AD16) after adipogenic induction in the absence (AD8, AD16) or presence of 5 mM NAD^+^ (AD8_NAD, AD16_NAD), 1 nM FK866 (AD8_FK866, AD16_FK866) or 50 mM EX527 (AD8_EX527, AD16_EX527). PCA was computed for the whole data. **(B–D)** Heatmap comparing expression from 660 genes DE exclusively in NAD^+^-treated cells (FDR-adjusted P value <0.05). **(C)** Overlap of DE transcripts between NAD^+^-treated cells and the rest of the tested conditions at day 8 and day 16 after adipogenic induction. **(D)** Heatmap comparing expression from 994 genes DE exclusively in NAD^+^-treated cells at both days 8 and 16 after adipogenic induction when compared with the rest of the samples. **(E–H)** Functional annotation of the 994 DE genes constituting the NAD^+^ transcriptional signature: biological processes (E and F) or KEGG pathways (G and H) for consistently upregulated (E and G) or downregulated (F and H) transcripts. **(I and J)** Homer de novo motif discovery analyses from promoters of genes specifically upregulated (I) or downregulated (J) after NAD^+^ treatment during adipogenic induction.

**Figure S2.**
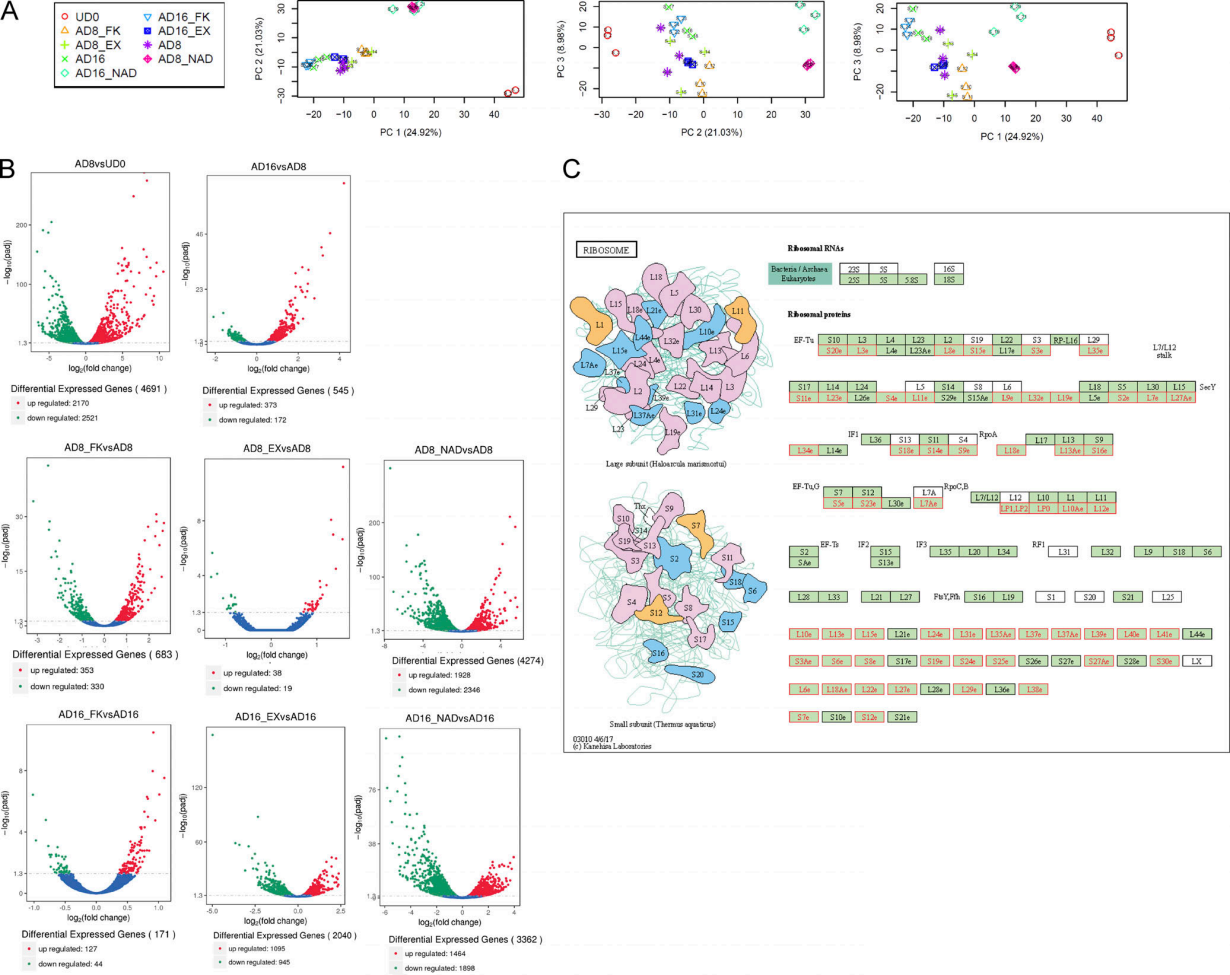
**RNA-seq data analyses. (A)** PCA were computed and plotted in two-dimensional PCA score plots, showing clustering of UD hMSC vs. differentiated cells (top), NAD^+^-treated (AD8_NAD, AD16_NAD) vs. untreated cells (middle), and day 8 (adipocyte commitment) vs. day 16 (terminally differentiated adipocytes; bottom). **(B)** Volcano plots show DE genes from the indicated comparisons (FDR-adjusted P value <0.05). **(C)** Illustration of the Ribosomal Pathway according to the KEGG. Ribosomal components are illustrated (left) and listed (right). Ribosomal proteins whose mRNA expression was consistently downregulated by NAD^+^ treatment during adipogenesis are highlighted in red.

To decipher the dynamic changes occurring in the transcriptome of hMSC during differentiation, we performed RNA sequencing (RNA-seq) analyses from undifferentiated hMSC, and differentiated cells at two different time points: at 8 d of adipogenic differentiation (middle of differentiation protocol) and at 16 d (end of differentiation), in untreated cells and in cells treated with NAD^+^, FK866, or EX527. We first performed an unbiased principal component analysis (PCA) revealing that, as expected, the largest variation was due to the differentiation process (PC1, 24.92%; Fig. 3 A and Fig. S2 A). Interestingly, the second component retaining 21.03% of the original variance between samples was mostly related to NAD^+^ treatment (Fig. 3 A and Fig. S2 A). Finally, a third component could be due to the progress of the differentiation process itself (PC3, 8.98%; Fig. 3 A and Fig. S2 A). We then performed differential gene expression analyses to compare between samples using DESeq2 (Love et al., 2014; see Materials and methods for details and Table S1 for normalized counts). These analyses further reinforced that NAD^+^ treatment had a large impact in the transcriptome, comparable to the differentiation process itself, as more than 3,000 genes were differentially expressed (DE) in differentiating cells treated with NAD^+^ when compared with their untreated controls (adjusted P < 0.05; Fig. S2 B). Concomitantly, inhibition of NAD^+^ biosynthesis by FK866 led to just 171 DE genes when compared with the untreated controls at the end of the adipogenic process (Fig. S2 B, AD16_FK vs. AD16). Hence, we sought to determine the molecular signatures of NAD^+^ treatment during adipogenic differentiation of hMSC. To do so, we first selected the transcripts specifically dysregulated by NAD^+^ treatment and found 660 common DE genes in NAD^+^ treated cells when compared with any other treatment or hMSC, which conform a unique NAD^+^ molecular signature (Fig. 3 B). Out of them, 157 genes were consistently upregulated, while 407 were always silenced by the treatment (Fig. 3 B and Table S2). Next, we sought to explore the molecular routes responsible for the impaired adipogenic capacity of NAD^+^-treated cells. Hence, we selected the genes which appeared consistently dysregulated in NAD^+^-treated cells in comparison with the rest of differentiating cells (AD, AD_EX, AD_FK). We identified 2,057 of these genes at day 8 and 1,969 at day 16 after adipogenic induction. 993 genes were shared, out of which 70% (703) were consistently downregulated, while 28% (279) were always overexpressed (Fig. 3, C and D; and Table S2). Gene ontology (GO) analyses revealed that most of the upregulated genes are implicated in apoptotic and response to stress processes while downregulated genes related to mRNA metabolism, cellular motility, and differentiation processes (Fig. 3, E and F; and Table S2). A subsequent pathway mapping in KEGG of the same transcripts revealed that many genes involved in steroid biosynthesis (*SQLE*, *DHCR7*, *FDFT1*, *SC5D*, *MSMO1*) were upregulated by NAD^+^ treatment during adipogenic differentiation of hMSCs. Also, apoptotic signaling pathways appeared active, as suggested by the overexpression of several death receptors (*TNFRSF-10A*, -*10B*, -*10D*), probably triggered by activated ER stress and unfolded protein response, evidenced by increased transcription of *IRE1α* (*ERN1*), BiP (*HSPA5*), and *XBP1* (Fig. 3 G and Table S2). Interestingly, increased cholesterol biosynthesis induces ER stress in macrophages (Feng et al., 2003). In contrast, a unique set of genes downregulated by an excess of NAD^+^ during adipogenic differentiation pertained to the ribosome pathway, involving many transcripts for ribosomal proteins (Fig. 3 H and Fig. S2 C), indicating that their expression is strongly suppressed by NAD^+^ in an adipogenic context. Additionally, many transcripts involved in cell adhesion and motility, which become expressed during differentiation, were downregulated in NAD^+^-treated cells, indicating that the adipogenesis process is arrested. Furthermore, we found that the JAK-STAT pathway was impaired in NAD^+^-treated cells, with downregulated transcripts including *STAT5A and STAT5B,* which are known positive regulators of the master adipogenic transcription factor PPARγ (Gurzov et al., 2016)*,* or the leptin encoding gene *LEP* (Fig. 3 H and Table S2). Taken together, these data point toward NAD^+^ promoting a proapoptotic and anti-adipogenic environment; therefore, inhibiting differentiation and maturation of adipocytes. Accordingly, a promoter screening for transcription factor binding motifs revealed significant enrichment for Suppressor of Mothers Against Decapentaplegic (SMAD) motifs within promoters of upregulated genes (Fig. 3 I; P = 10^−7^), and for CEBP:AP1 motifs amongst downregulated genes’ promoters (Fig. 3 J; P = 10^−8^). Indeed, SMADS are well-known apoptotic regulators, while CEBP is a master adipogenic transcription factor (Conery and Luo, 2006; Lefterova et al., 2008).

### SIRT1 regulates terminal differentiation of pre-adipocytes but is dispensable for adipogenic commitment

**Figure 4.**
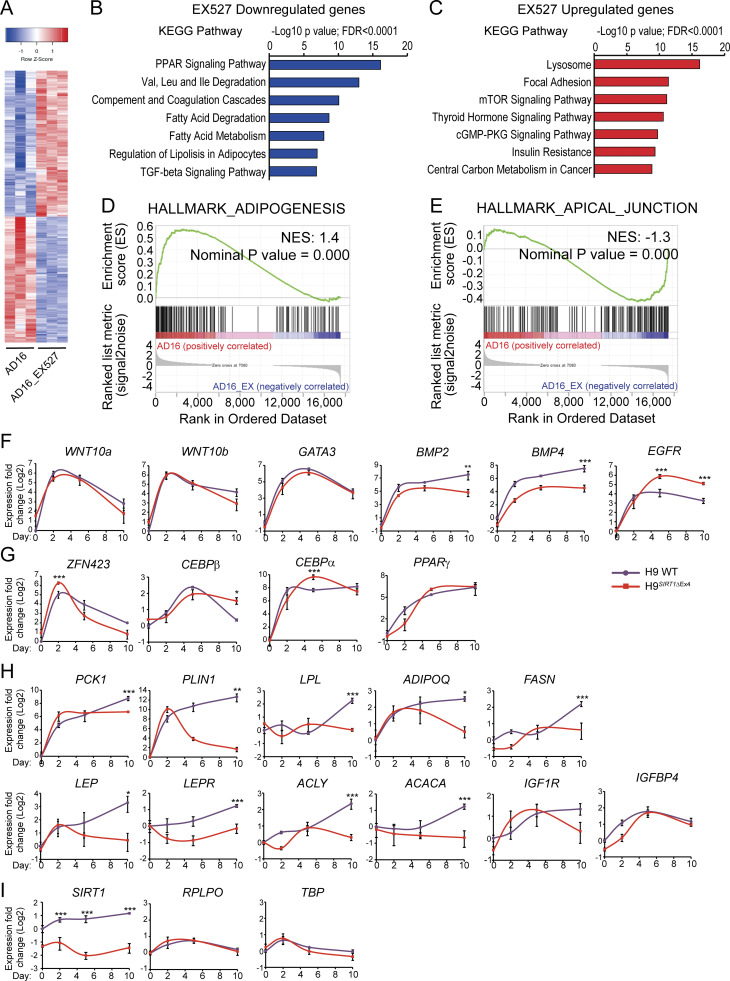
**SIRT1 activity is essential for terminal differentiation of pre-adipocytes. (A)** Heatmap comparing expression from 2,040 genes DE between EX527-treated cells (50 mM) and untreated cell at day 16 after adipogenic induction on hMSC. **(B and C)** FDR-adjusted P value <0.05. KEGG pathway enrichment analyses from genes downregulated (B) or upregulated (C) by EX527 treatment during adipogenic differentiation, at day 16 after induction, compared with untreated, terminally differentiated adipocytes. **(D and E)** GSEA investigated within the MSigDB “Hallmark” gene set collection. Genes were rank-ordered by differential expression between terminally differentiated adipocytes untreated (AD16) or treated with EX527 (AD16_EX). **(F–I)** Gene expression from H9 WT and H9^*SIRT1*ΔEx4^ cells was measured by qRT-PCR at days 0, 2, 5, and 10 after adipogenic induction. Genes involved in pre-adipocyte commitment (F), PPARg expression (G), lipid metabolism and adipocyte markers (H), and control genes (I) were analyzed from three biological replicates. Relative expression to *Tbp* or *Hrpt* housekeeping genes was obtained and normalized to H9 at day 0 = 1. Data were converted to Log_2_ scale and fold change is presented. Graphs show mean ± SEM (*P < 0.05, **P < 0.01, ***P < 0.001, two-way ANOVA with Bonferroni’s post-test).

**Figure S3.**
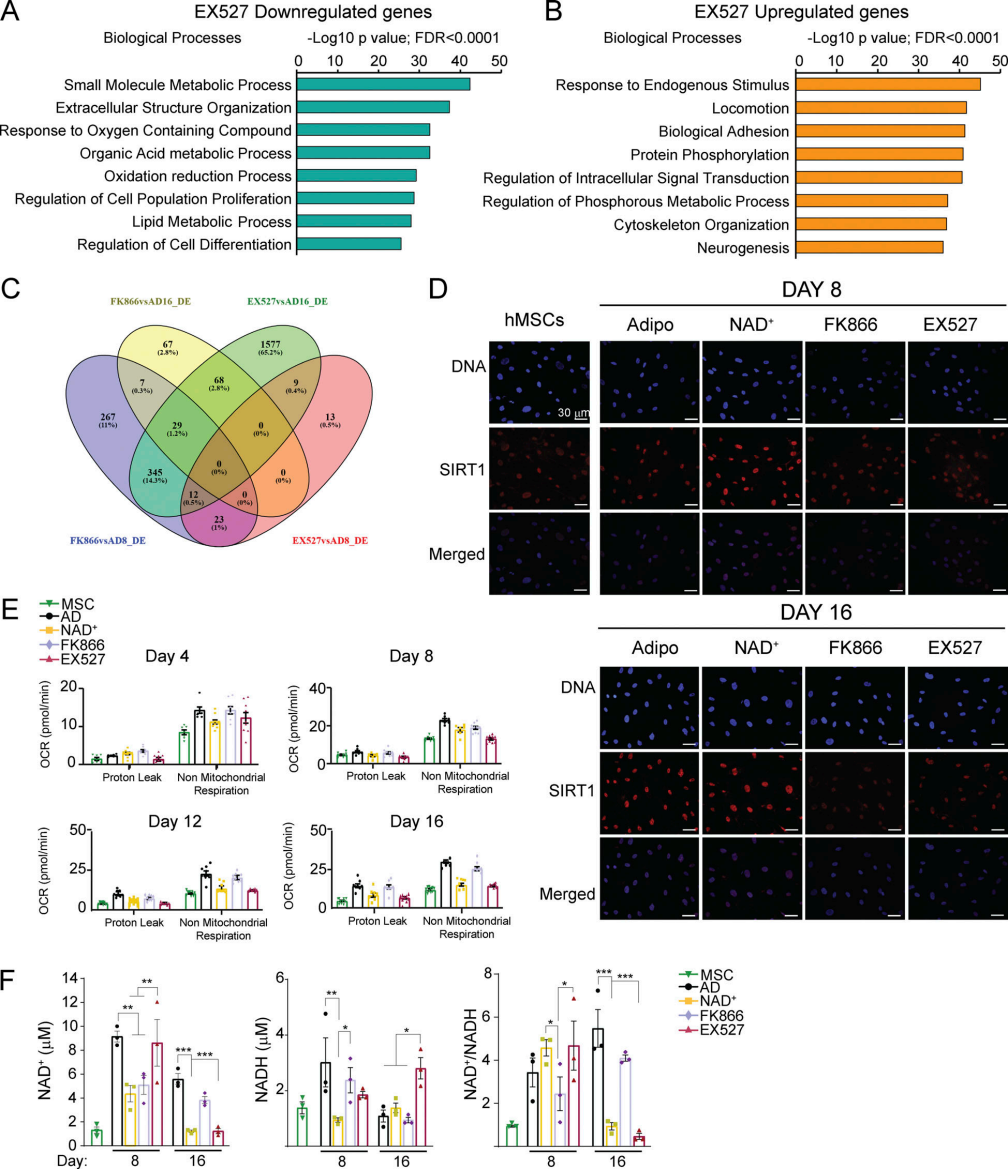
**Transcriptional and metabolic rewiring during adipogenic differentiation triggered by SIRT1 or NAMPT inhibition. (A and B)** Biological processes enrichment analyses from genes downregulated (A) or upregulated (B) by EX527 treatment during adipogenic differentiation, at day 16 after induction, compared with untreated, terminally differentiated adipocytes. **(C)** Venn diagram shows overlapping DE genes between indicated comparisons: FK866vsAD8_DE and FK866vsAD16_DE: mRNA was analyzed from cells during adipogenic differentiation (day 8 or day 16) from untreated (AD) or treated with 1 nm FK866 during differentiation. EX527vsAD8_DE and EX527vsAD16_DE: mRNA was analyzed from cells during adipogenic differentiation (day 8 or day 16) from untreated (AD) or treated with 50 mM EX527 during differentiation. **(D)** SIRT1 protein levels and subcellular localization were analyzed by immunofluorescence at days 8 and 16 after adipogenic induction on hMSC. Cells were either untreated (Adipo) or treated with the indicated compounds. *n* = 2 biological and 7 technical replicates. Scale bars represent 30 μm. **(E)** Mitochondrial bioenergetic parameters calculated from extracellular flux analyses: Proton leak and non-mitochondrial respiration. **(F)** Total NAD^+^ and NADH were measured at days 8 and 16 after adipogenic induction in hMSCs from three biological replicates. Data are represented by mean ± SEM. Two-way ANOVA followed by Tukey’s post-test. *P < 0.05, **P < 0.01, ***P < 0.001. AD, adipogenic-induced cells; NAD^+^, adipogenic-induced cells treated with 5 mM NAD^+^; FK866, adipogenic-induced cells treated with 1 nM FK866; EX527, adipogenic-induced cells treated with 50 mM EX527; MSC, untreated, undifferentiated hMSC.

If an excess of NAD^+^ hinders the adipogenic transcriptional reprogramming, while inhibiting NAD^+^ biosynthesis has only a mild transcriptional effect at the end of the adipogenic process, it is conceptually apparent that critical NAD^+^ consuming enzymes such as SIRT1 would not be essential for adipocyte differentiation. Accordingly, SIRT1 inhibition by EX527 had only a mild effect on the transcriptome at day 8, with just 57 DE genes compared with the untreated cells (Fig. S2 B, AD8_EX vs. AD8; Table S3). Strikingly, in terminally differentiated adipocytes, we found 2,040 DE genes, with 1,095 upregulated and 945 downregulated transcripts (Fig. S2 B, AD16_EX vs. AD16; Fig. 4 A; and Table S3). This is in line with the absence of lipid accumulation in these cells and indicates that SIRT1 is probably dispensable for lineage commitment, but it is still essential for terminal differentiation of adipocytes. Interestingly, at day 8 of differentiation, the anti-adipogenic genes *EGR1* and *NR4A1* (Boyle et al., 2009; Zhang et al., 2018) were overexpressed in EX527-treated cells (Table S3; P < 0,0001, fold change >2). These genes have previously been shown to be upregulated in the early mitotic clonal expansion phase during preadipocyte differentiation (Zhang et al., 2018), further reinforcing that SIRT1 inhibition compromises maturation, but not commitment of preadipocytes. Accordingly, at day 16, many genes pertaining to pathways such as fatty acid metabolism, degradation, or lipolysis were downregulated in cells treated with EX527, most of them implicated in PPAR signaling (Fig. 4 B, Fig. S3 A, and Table S3). Amongst these, the adipokine genes leptin (*LEP*), leptin receptor (*LEPR*), and adiponectin (*ADIPOQ*) showed significantly lower levels in EX527-treated cells (Table S3). Interestingly, SIRT1 inhibition upregulated transcripts enriched for adhesion and locomotion, critical processes during the onset of the adipogenesis, including the fibronectin gene *FN1*, which inhibits adipocyte maturation (Liu et al., 2005; Fig. 4 C, Fig. S3 B, and Table S3). Accordingly, gene set enrichment analysis (GSEA) identified adipogenesis and apical junction as the top-ranked hallmarks enriched in untreated and EX527-treated cells, respectively (Fig. 4, D and E). Together, these data indicate that SIRT1 activity is essential for adipogenic differentiation, specifically by tightly controlling the transition between adipocyte commitment and maturation, and the maturation process itself. To confirm this, we explored adipogenesis-related transcripts in H9 and H9^*SIRT1*ΔEx4^ cells at days 0, 2, 5, and 10 after adipogenic induction. We first studied expression from genes involved in the adipogenic commitment, which is mediated by members of the BMP and Wnt families (Tang and Lane, 2012). As reported (Cawthorn et al., 2012; Christodoulides et al., 2009), *WNT10a* and *WNT10b* and *GATA3* genes increased during commitment and decreased at later differentiation stages to comparable levels in both genotypes, while significant differences in *BMP2* and *BMP4* expression appeared at terminal differentiation (Fig. 4 F). The early adipogenesis marker *EGFR* remained significantly high in H9^*SIRT1*ΔEx4^ cells, which might oppose progression into mature adipocytes (Harrington et al., 2007). We also explored the transcription factors controlling *PPAR*γ expression *ZFN423*, *CEBPβ*, and *CEBPα* (Gupta et al., 2010; Rosen et al., 2002), finding that all these were highly expressed in both cell lines, and accordingly, *PPAR*γ gene expression levels and dynamics appeared similar (Fig. 4 G). Interestingly, in H9^*SIRT1*ΔEx4^ cells, *CEBPβ* expression was higher than in WT cells at terminal differentiation, reinforcing the idea that adipogenic progenitors do not progress into adipocytes (Fig. 4 G). Finally, expression analyses of genes involved in fatty acid metabolism and PPARγ signaling revealed that these transcripts significantly increased specifically in WT cells at terminal differentiation (day 10, Fig. 4 H), and paralleling our observations in EX527-treated hMSCs, H9^*SIRT1*ΔEx4^ cells do not show high expression of these adipocyte markers (Fig. 4 H). As expected, *SIRT1* expression remained low in H9^*SIRT1*ΔEx4^ cells, while no significant expression changes were detected in the housekeeping genes *RPLPO* and *TBP* (Fig. 4 I).

**Figure 5.**
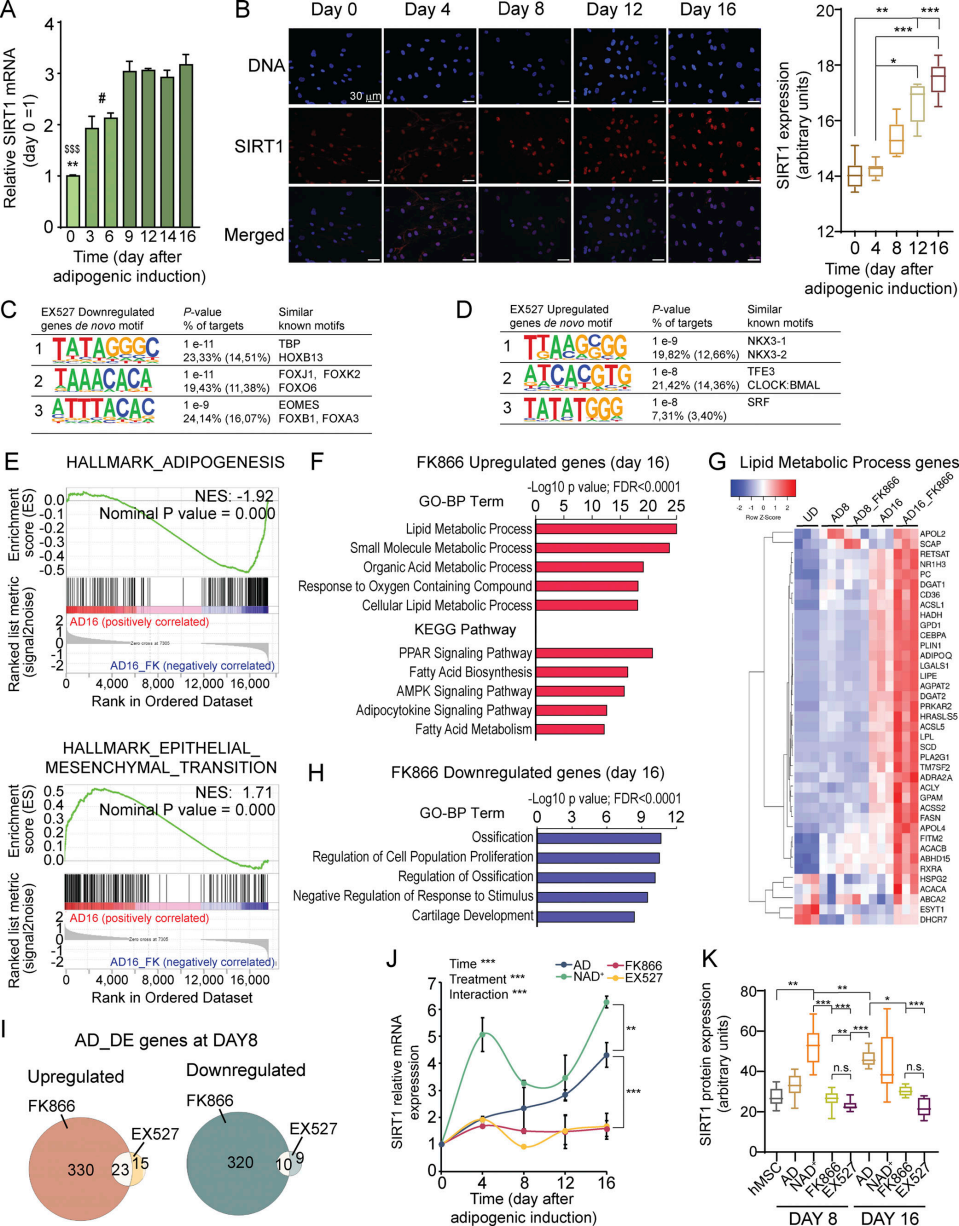
**The NAD**^**+**^ **salvage pathway is dispensable for adipogenesis. (A)** *SIRT1* gene expression levels were assessed by RT-qPCR at the indicated days after adipogenic induction on hMSC. *n* = 3 biological and 2 technical replicates. One-way ANOVA followed by Tukey’s post-test. *P < 0.05, **P < 0.01, ***P < 0.001. Symbol key for multiple comparisons: *: day 0 vs. days 3, 6; $: day 0 vs. days 9–16; #: days 3, 6 vs. days 9–16. **(B)** SIRT1 protein expression and subcellular location were explored by immunofluorescence at the indicated days after adipogenic induction on hMSC. Scale bars represent 30 μm. Boxplot shows densitometric analyses from *n* = 2 biological and 7 technical replicates. Kruskal-Wallis test followed by Dunn’s multiple comparisons test was applied. *P < 0.05, **P < 0.01, ***P < 0.001. **(C and D)** Homer de novo motif discovery analyses from promoters of genes downregulated (C) or upregulated (D) by EX527 treatment during adipogenic induction, at terminal differentiation (day 16). **(E)** GSEA was investigated within the MSigDB “Hallmark” gene set collection. Genes were rank-ordered by differential expression between terminally differentiated adipocytes untreated (AD16) or treated with 1 nM FK866 (AD16_FK). **(F)** Functional annotation (biological processes [BP] and KEGG pathways) for 144 genes upregulated by FK866 treatment during adipogenesis, at terminal adipogenic differentiation (day 16). **(G)** Heatmap comparing expression levels between the indicated samples at day 16 from 39 genes involved in lipid metabolism. **(H)** Biological processes enriched in 44 genes downregulated by FK866 treatment during adipogenesis, at terminal differentiation (day 16). **(I and J)** Overlap of DE genes (up- or downregulated) comparing EX527 and FK866-treated cells during adipogenic induction, at day 8. **(J)** *SIRT1* gene expression levels were assessed by RT-qPCR at the indicated days after adipogenic induction on hMSC either untreated (AD) or treated with the indicated drugs. *n* = 3 biological and 2 technical replicates. One-way ANOVA followed by Tukey’s post-test. **P < 0.01, ***P < 0.001. **(K)** Boxplot showing SIRT1 protein levels analyzed by immunofluorescence at days 8 and 16 after adipogenic induction on hMSC. Cells were either untreated (AD) or treated with the indicated compounds. Densitometric analyses are from *n* = 2 biological and 7 technical replicates. Kruskal-Wallis test followed by Dunn’s multiple comparisons test was applied. *P < 0.05, **P < 0.01, ***P < 0.001.

Remarkably, although SIRT1 mRNA expression increased at the beginning of adipogenic induction (Figs. 4 I and 5 A), the protein significantly increased after 8 d of adipogenic induction (Fig. 5 B; P < 0,0001, Kruskal-Wallis with Dunn’s post-test), probably due to post-transcriptional regulation, and further reinforcing the notion that SIRT1 is essential for adipocyte maturation. Strikingly, a motif analysis revealed that pharmacological inhibition of SIRT1 downregulated preferred targets genes for distinct members of the FOX family of transcription factors (Fig. 5 C; P = 1E−11) while overexpressed genes were enriched for motifs binding homeobox (NKX3-1, -2) or b-Helix-loop-helix transcription factors such as the circadian master regulator CLOCK:BMAL1 (Fig. 5 D; P = 1E−8), which is tightly controlled by SIRT1 activity (Nakahata et al., 2008).

SIRT1 activity heavily relies on NAD^+^ availability, hence the treatment with the NAMPT inhibitor FK866, which is known to dampen intracellular NAD^+^ levels, would prevent SIRT1 activation and the subsequent adipocytic gene expression program. Surprisingly, FK866 treatment during adipogenesis led to enhanced adipocytic differentiation, through overexpression of adipogenic hallmarks, increased adipocytokine, and PPAR signaling over the control, NT adipocytes, at terminal differentiation (Fig. 5, E and F; Fig. S3 C; and Table S4). Indeed, 39 genes involved in lipid metabolism, including *CD36*, *CEBPA*, *ADIPOQ*, *LPL*, *ACLY*, *FASN*, *ACACA*, *ACACB*, *PLIN1*, and *RETSAT*, were highly expressed in terminal adipocytes treated with FK866 (Fig. 5 G). Moreover, genes related to ossification or cartilage development were downregulated in these cells (Fig. 5 H), indicating that low NAD^+^ levels induced by FK866 treatment potentiate the adipogenic over the osteogenic lineage in hMSCs (Chen et al., 2016). These results are in line with our previous observation that NAD^+^ treatment hinders the differentiation of hMSC into adipocytes; yet, they are in contrast with the need for SIRT1 activity, which relays on its cofactor NAD^+^, for adipocyte maturation. Furthermore, out of 57 DE genes between the control and EX527-treated cells at day 8 of differentiation, 33 (58%) were also dysregulated by FK866 treatment, suggesting that SIRT1 is not active in FK866-treated cells at day 8 (Fig. 5 I). Accordingly, SIRT1 expression both at the mRNA and protein levels was dampened by EX527 and FK866 to similar levels during adipogenic induction (Fig. 5 J, two-way ANOVA with Tukey’s post-test; Fig. 5 K, Kruskal-Wallis with Dunn’s post-test; Fig. S3 D). These data suggest that SIRT1 function might be dispensable for adipocyte commitment, but it is necessary for differentiation and increased levels of NAD^+^ essential for SIRT1 activity do not rely on the NAM/NMN salvage pathway to NAD^+^.

### Increased NAD^+^ bioavailability during the adipogenic process impairs the rise of mitochondrial respiration capacity in hMSCs

**Figure 6.**
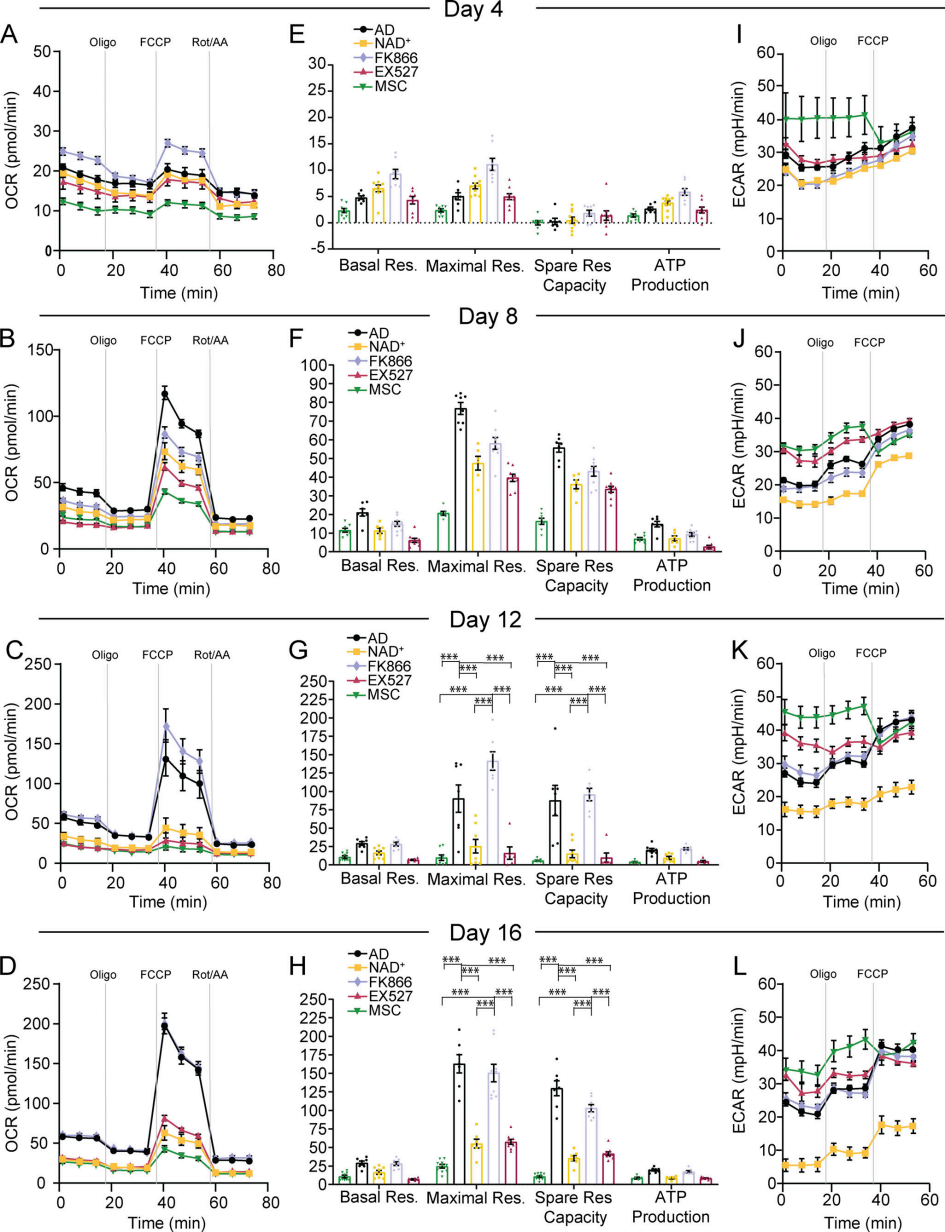
**NAD**^**+**^ **impairs mitochondrial bioenergetics during adipogenic induction in hMSC.** Analysis of OCR and ECAR was performed using Seahorse XF analyzer to assess mitochondrial respiration and lactate production from *n* = 3 biological replicates with 6–10 technical replicates each. **(A–D)** OCR was measured at days 4 (A), 8 (B), 12 (C), or 16 (D) after adipogenic induction in hMSC, in the absence or presence of the indicated treatments. With sequential addition of oligomycin (Oligo; complex V inhibitor), FCCP (a protonophore), and Rotenone/antimycin A (Rot/AA; complex III inhibitor). **(E–H)** Mitochondrial bioenergetic parameters were calculated from extracellular flux analyses: basal respiration, maximal respiratory capacity, spare respiratory capacity, and ATP production at the indicated days after adipogenic induction. Two-way ANOVA followed by Tukey’s post-test. ***P < 0.001. **(I–L)** ECAR was measured after serial addition of oligomycin and FCCP. Data is presented by mean ± SEM. AD: adipogenic-induced cells; NAD^+^, adipogenic-induced cells treated with 5 mM NAD^+^; FK866, adipogenic-induced cells treated with 1 nM FK866; EX527, adipogenic-induced cells treated with 50 mM EX527; MSC, untreated, undifferentiated hMSC.

A major shift in metabolic phenotype is a hallmark of adipogenic differentiation. Hence, we investigated the functional effect of altering NAD^+^ balance and SIRT1 activity during hMSC adipogenesis on energy metabolism by performing extracellular flux analysis for measuring cellular bioenergetics. As expected, NT hMSCs progressively increased their respiratory capacity during the adipogenic differentiation when compared to undifferentiated hMSCs, which retained low oxygen consumption rates across all tested days (Fig. 6, A–D). We observed that at day 4, all tested conditions retained low respiratory capacity, comparable to undifferentiated cells (Fig. 6 A), while the most prominent increase in respiration capacity occurs between days 8 and 16 (Fig. 6, B–D). Indeed, FK866 treatment overall allowed the metabolic reprogramming during adipogenesis of hMSCs; however, NAD^+^ treatment obstructed the progressive increase in mitochondrial respiration (Fig. 6, A–D). Interestingly, pharmacological inhibition of SIRT1 by EX527 showed major effect after day 12, consisting of markedly reduced respiratory capacity compared to the untreated cells. These results confirm that metabolic reprogramming during adipogenesis is compromised by SIRT1 inhibition and reinforce the notion that SIRT1 is essential for adipocyte maturation. We observed major differences between treatments in maximal respiration and spare respiratory capacity at days 12 and 16, when induced cells either untreated or treated with FK866 showed a very significant increase compared to the rest of the conditions (Fig. 6, E–H; P < 0.0001, two-way ANOVA with Tukey’s post-test), indicating a high rate of oxidative phosphorylation in these cells. Non-mitochondrial respiration and proton leak did not show significant differences at any of the studied conditions (Fig. S3 E). Notably, extracellular acidification rate (ECAR) measurements revealed that undifferentiated hMSCs exhibit a glycolytic phenotype while treatment with NAD^+^ during adipogenic induction also diminished the glycolytic flux (Fig. 6, I–L), indicating that these cells are metabolically less active.

**Figure 7.**
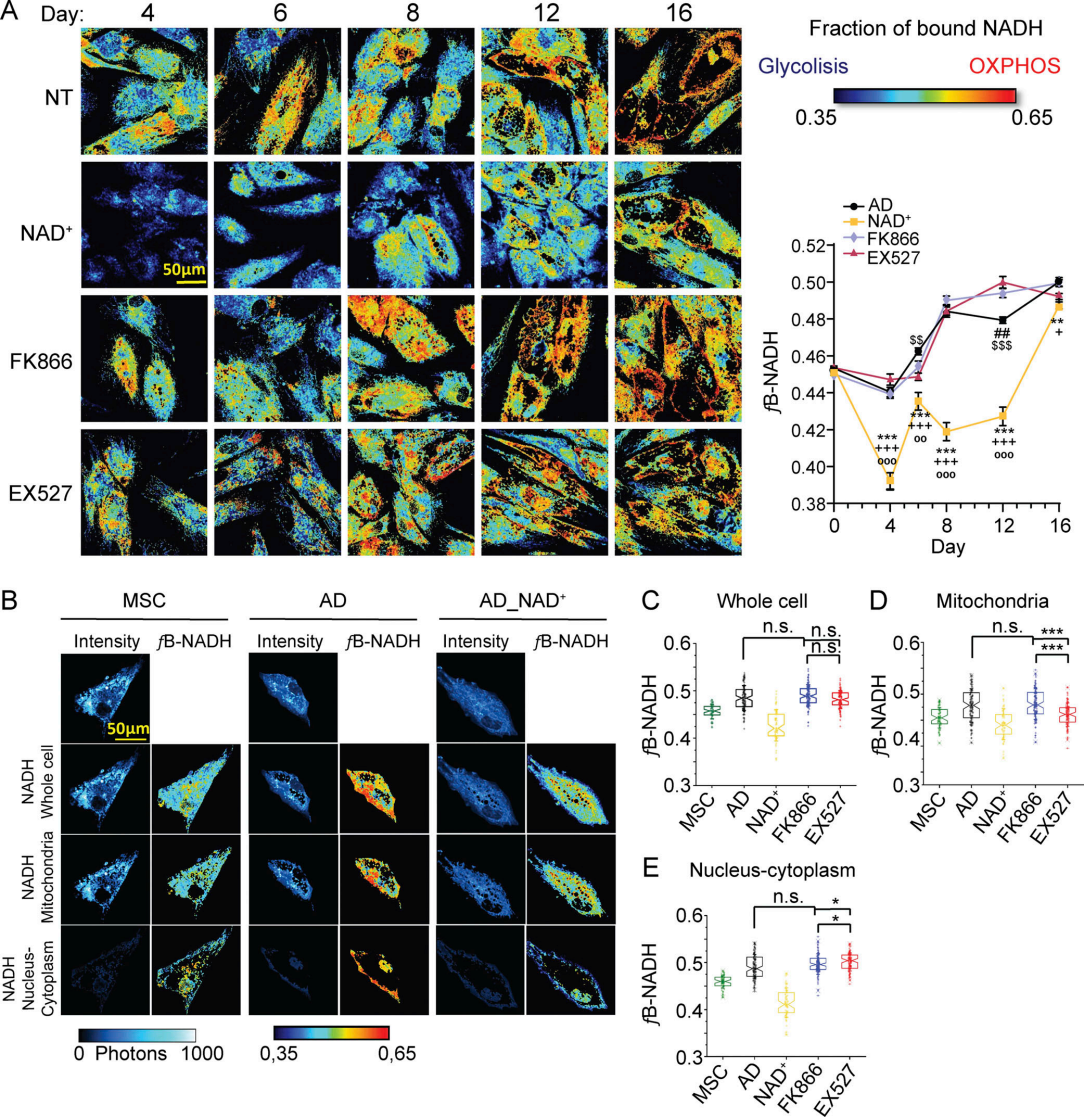
**Subcellular compartmentalization of NADH metabolism during adipogenesis depends on SIRT1 activity. (A)** Representative images of fB_NADH of hMSCs during adipogenic differentiation at days 4, 6, 8, 12, and 16 of induction in the absence (NT) or presence of the indicated treatments: 5 mM NAD^+^, 1 nM FK866, or 50 μM EX527. Low fB_NADH (blue colors) corresponds to a cellular glycolytic phenotype, while high fB_NADH (red colors) corresponds to an OXPHOS phenotype. Quantification of fraction of bound NADH in each culture condition was performed from *n* = 14 single cells. Experiments were conducted per triplicate. Data are presented by mean ± SEM. * AD vs. NAD^+^; $ AD vs. EX527; # AD vs. FK866; + NAD^+^ vs. FK866; ° NAD^+^ vs. EX527. **(B)** Representative images of intensity and fB_NADH of hMSCs, pre-adipocytes (AD), and cells treated with NAD^+^ during adipogenic induction (AD_NAD^+^) imaged at day 8 of differentiation, show different spatial distributions of fraction of bound NADH in different cell compartments such as mitochondria, nucleus, and cytoplasm. **(C–E)** Quantification of fB_NADH in single cells at day 8 from hMSC (MSC), pre-adipocyte (AD), and cells treated with the indicated compounds during adipogenic differentiation. Quantification from *n* = 63–125 cells was performed in the whole cell (C), or the mitochondrial (D) and nuclear/cytoplasmic (E) subcellular compartment. Two-way ANOVA followed by Tukey’s post-test. *P < 0.05, **P < 0.01, ***P < 0.001.

**Figure S4.**
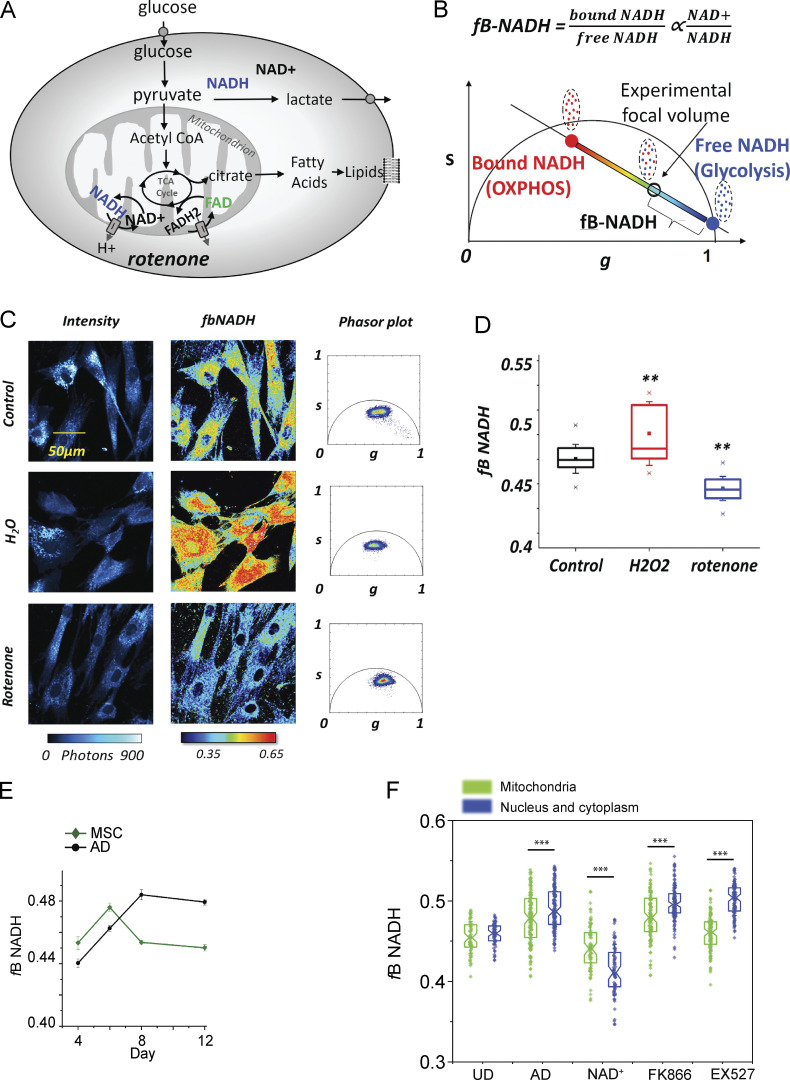
**Metabolic trajectories assessed by 2P-FLIM on NADH. (A)** Schematic representation of cellular metabolism. Glucose breakdown through glycolysis and the TCA cycle generates reduced NADH and FADH2. Quiescent cells have a basal rate of glycolysis, converting glucose to pyruvate, which is then oxidized in the TCA cycle. As a result, the majority of ATP is generated by OXPHOS. Non-proliferating, differentiated cells are characterized by a low NADH/NAD^+^ ratio. During proliferation, the large increase in glycolytic flux rapidly generates ATP in the cytoplasm. Most of the resulting pyruvate are converted into lactate by lactate dehydrogenase A, which regenerates NAD^+^ from NADH. Proliferating cells are characterized by a high NADH/NAD^+^ ratio. Rotenone blocks the respiratory chain through complex I while H2O2 increase the NAD^+^:NADH ratio. **(B)** Metabolic trajectory between free NADH and bound NADH indicates a shift from a glycolytic to a OXPHOS cellular phenotype as free/bound NADH ratio corresponds to NAD^+^:NADH ratio. The fB_NADH of the experimental point is graphically calculated from the location of free NADH. **(C)** Representative images of intensity, fB_NADH, and Phasor plot of hMSCs with different treatments: control, rotenone (respiratory chain inhibitor), and H_2_O_2_ (induces oxidative stress). Accumulation of reduced NADH by blocking the respiration chain shifts the cellular metabolic signature toward free NADH, while oxidative stress shifts the cellular metabolic signature toward bound NADH. **(D)** Quantification of fraction of bound NADH in an ROI with different metabolic treatments. One-way ANOVA followed by Tukey’s post-test. **P < 0.01. **(E)** Quantification of fraction of bound NADH during adipogenic differentiation at with (black) or without adipogenic culture medium (dark green). **(F)** Data are presented as mean ± SEM. Quantification of fB_NADH in mitochondria (green) and in nucleus/cytoplasm (blue) in single cells at day 8 of adipogenic differentiation in the absence (AD) or in the presence of the indicated treatments. hMSC (UD) were also assessed. *n* = 63–125 cells; ***P < 0,001, Student’s *t* test.

**Figure S5.**
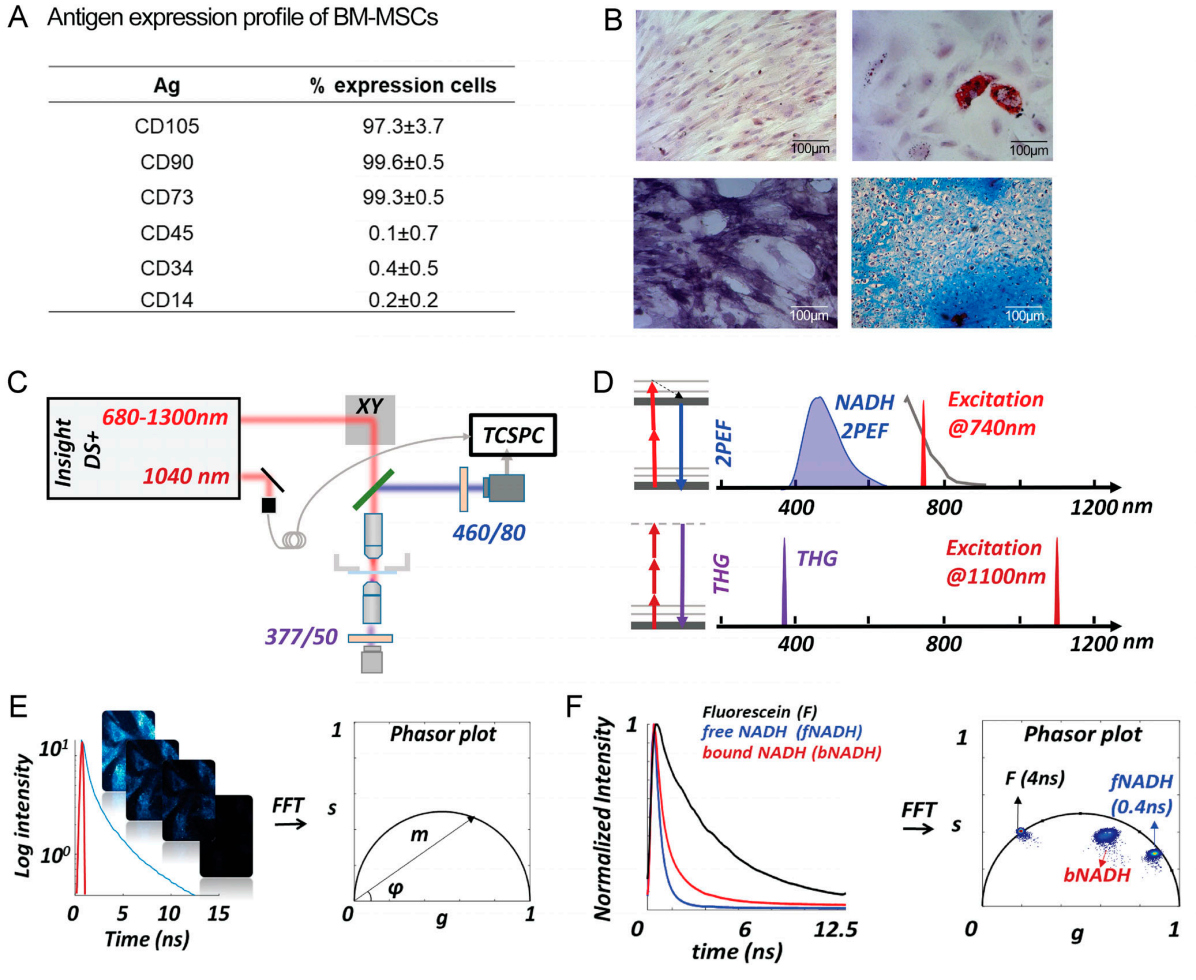
**hMSC characterization and experimental setup. (A)** Expression cell markers in MSCs was determined by flow cytometry; data correspond to mean percentage of cells positive to each marker ± SD, *n* = 3 biological replicates. **(B)** hMSCs stained with toluidine blue (top left); adipogenic differentiation was determined by the presence of lipid vacuoles positive to ORO (top right); osteogenic differentiation was determined by alkaline phosphatase assay (bottom left); and chondrogenic differentiation was assessed by matrix positive to alcian blue in cryosections of micromasses (bottom right). *n* = 3 biological replicates. **(C)** Scheme of the experimental setup used for this work. **(D)** Principles of two-photon excitation fluorescence (2PEF) and THG. Two-photon excitation of NADH is performed at 740 nm with emission collected with band-pass filters centered at 460 nm, which resulted primarily from NADH. THG is performed at 1,100 nm, and the signal collected with a band-pass filter centered at 377 nm. **(E)** The multi-exponential fluorescence intensity decay in every pixel of the image is transformed with an FFT; the real (*g*) and imaginary (*s*) parts are plotted in the graphical Phasor plot. **(F)** Example of fluorescence intensity decay of fluorescein and free and bound NADH in solution and their locations in the Phasor plot.

Attempting to relate intracellular NAD^+^ bioavailability to the observed changes in adipogenic capacities and metabolic phenotypes of the cells under different treatments, we used a colorimetric assay to measure total NAD^+^ and NADH (Fig. S3 F). Unexpectedly, we found that hMSCs cells treated with NAD^+^ during adipogenic induction presented lower intracellular NAD^+^ and NADH levels than control cells, while the EX527-treated cells appeared comparable to control cells at day 8, yet their redox ratio was very significantly altered at day 16. Importantly, total measurements of NAD^+^ and NADH pose the limitation that they do not inform about the distribution of the cofactors in distinct subcellular compartments or about their function (i.e., whether they are being used for reactions or free inside the cells). Hereby, we performed label-free 2P-FLIM of the intrinsic metabolic biomarker NADH in live cells (see Materials and methods section). We measured the fraction of bound NADH (fB_NADH) during adipogenic differentiation with all treatments with a micrometer pixel resolution (Fig. 7 A) to quantify the redox ratio (NAD^+^/NADH) and the relative contribution of oxidative phosphorylation (OXPHOS) to glycolysis (Fig. S4, A–D; Bird et al., 2005; Stringari et al., 2011; Ung et al., 2021) .The results were in agreement with the extracellular flux analyses, and we observed that the fB_NADH progressively increased during adipogenic differentiation, as a result of the metabolic shift from a glycolytic to an OXPHOS phenotype (Fig. 7 A and Fig. S5, A–E). Also, NAD^+^ treatment consistently induced low fB_NADH across all tested days (Fig. 7 A), reinforcing the notion that increased NAD^+^ bioavailability during adipogenic differentiation opposes the metabolic shift toward OXPHOS. With this approach, we only found a significant reduction of fB_NADH in cells treated with EX572 at the end of the differentiation process (Fig. 7 A), which is in line with the extracellular flux analyses and total NAD^+^ and NADH measurements, indicating that SIRT1 inhibition hinders adipocytic maturation.

### Subcellular compartmentalization of NADH metabolism during adipogenesis depends on SIRT1 and is impaired by abnormally high NAD^+^ levels

Our FLIM and extracellular flux analyses showed important disparities, specifically for the cells treated with EX527 during adipogenic differentiation. Interestingly, at days 8 and 12, two photon-FLIM did not show significant differences between EX527 and untreated cells, while extracellular flux analyses indicated an important reduction in respiratory capacity of EX527-treated cells after day 8. For these reasons, we sought to determine the metabolic signatures at distinct subcellular compartments, in order to dissect the subcellular location of the metabolic changes in these cells using two-photon FLIM (see Materials and methods section and Fig. S1). This technique allowed us to capture intensity images and maps of fB_NADH in the entire cell and different cellular compartments such as mitochondria and nucleus/cytoplasm, as shown in Fig. 7 B for an hMSC, and at day 8 after adipogenic induction in untreated (AD) or NAD^+^-treated cells (AD_NAD^+^). Our single-cell analysis at day 8 showed a wide distribution of cellular metabolic states in every culture condition (undifferentiated [UD], AD, AD-NAD^+^, AD-FK866 or AD-EX527; Fig. 7 D). Considering the metabolic fingerprint of the entire cell, NAD^+^-treated cells showed lower fB_NADH than untreated cultures, while FK866 and EX527 treatments did not show differences with untreated cells (two-way ANOVA with Tukey’s post-test). Interestingly, we observed distinct subcellular compartmentalization of NADH metabolism depending on the treatment (Fig. 7, D and E). Consistently, fB_NADH at both mitochondria and nucleus/cytoplasm were lower in NAD^+^-treated cells (Fig. 7, D–E). Moreover, the fB_NADH in the cytoplasm/nucleus was higher than in mitochondria in differentiating cells, while remained similar in hMSC; yet, NAD^+^ treatment showed an opposite ratio, where the mitochondrial fraction showed significantly higher levels of fB_NADH (Fig. S4 F). Interestingly, the fB_NADH of mitochondria appeared significantly decreased in cells treated with the SIRT1 inhibitor, while the fB_NADH increased within the nucleus/cytoplasm compartment in these cells when compared with the NT or with the FK866-treated cells (Fig. 7, D and E; P < 0,05, two-way ANOVA with Tukey’s post-test). Together, out data reveal that energy metabolism progress during adipogenic differentiation is impeded by both SIRT1 inhibition and high NAD^+^ levels, through different mechanisms at distinct subcellular compartments. These observations have further implications for disease, as provide previously unappreciated subcellular insights into the previously reported efficacy of NAD^+^ as a treatment for diet-induced obesity and metabolic dysfunction (Connell et al., 2019; Yoshino et al., 2018).

## Discussion

In this study, we have demonstrated a previously unappreciated role for NAD^+^-SIRT1 interplay in adipogenesis from hMSC which is dependent on the differentiation stage. We have shown that SIRT1 activity is essential for terminal adipocyte differentiation, and unexpectedly, NAD^+^ availability fine tunes the adipogenic process. Previous research demonstrates that SIRT1 inhibits adipogenesis in MSCs (Bäckesjö et al., 2006; Li et al., 2011; Peltz et al., 2012; Puri et al., 2012; Song et al., 2019; Tseng et al., 2011; Zhou et al., 2015); however, the dynamics of SIRT1 activity during adipogenesis remains poorly understood. Our gene expression data reveal that SIRT1 activity is dispensable for adipocyte commitment, as out of ∼5,000 DE genes at day 8 of differentiation, <60 were significantly altered between normally differentiating cells and those with inhibited SIRT1 activity. Unexpectedly, transition to mature adipocytes was strongly dampened by SIRT1 inhibition, demonstrating that SIRT1 is essential. These results are in line with the notion that reducing SIRT1 activity specifically in fat could improve metabolic function in obesity (Mayoral et al., 2015). Moreover, in mice lacking SIRT1 specifically in MSC, their adipogenic capacity appears compromised leading to significant reduction in subcutaneous fat (Simic et al., 2013), further reinforcing this notion.

We have described a PPARγ isoform switch upon NAD^+^ or β-NMN treatment, which is particularly apparent at day 8 of adipogenic induction (Fig. 2, D–F). Differential chromatin dynamics at *PPAR*γ1 and *PPAR*γ2 promoters might underlie this effect, through specific epigenetic mechanisms impacted by NAD^+^. For example, it has been shown that C/EBPα is recruited to the *PPAR*γ2 promoter and directly transactivates its expression (Clarke et al., 1997; Elberg et al., 2000), while *PPAR*γ1 can be expressed in the absence of C/EBPα (Hamm et al., 2001). Hereby, it appears reasonable that specific epigenetic mechanisms differentially affected by NAD^+^ levels might impose the isoform switch (Lee and Ge, 2014). However, the functional differences of the two PPARγ isoforms in adipogenesis remain largely unknown, and further studies are needed to disentangle this isoform-specific regulation.

Several studies report that the redox state of stem cells is tightly regulated during differentiation, and thus activation of oxidative pathways represents a metabolic signature of stem-cell differentiation (Folmes et al., 2012; Folmes et al., 2011). Along the same lines, stem cells appear to contain lower levels of ROS than their mature progeny, and that ROS accumulation triggers intracellular signaling required for differentiation (Ito et al., 2004; Smith et al., 2000; Tsatmali et al., 2005; Yanes et al., 2010). Hence, it appears that metabolic reprogramming activates specific signaling cascades promoting either stem-cell maintenance and self-renewal (reduced state) or proliferation and differentiation (oxidized state). Here, we have shown that fueling energy metabolism with NAD^+^ markedly obstructs adipogenic differentiation. Notably, it was previously demonstrated that NAD^+^ treatment in cultured cells rapidly shifts the intracellular redox state and stabilizes NAD^+^-consuming enzymes in the nucleus (Aguilar-Arnal et al., 2016; Han et al., 2011; Wilk et al., 2020). Hence, by imposing an oxidative redox state during early adipogenesis through NAD^+^ treatment, a new gene expression program emerges and contributes to translational arrest and induction of proapoptotic pathways, hereby these cells acquire a quiescent metabolic phenotype (Figs. 2 and 4). NAD^+^ levels rise at late stages of differentiation (Okabe et al., 2020), probably as a result of the increased oxidative metabolism, which in our hMSC adipogenic differentiation model rapidly emerges between days 8 and 12 (Figs. 6 and 7 A). This is coincident with increased levels of SIRT1 transcription and protein expression (Fig. 5, J and K), reinforcing the idea of a SIRT1-dependent late stage of adipogenic differentiation. At this regard, obstructing the NAD^+^ salvage pathway through constant inhibition of the rate-limiting enzyme NAMPT during adipogenesis did not hinder the adipogenic capacity, demonstrating that the metabolic switch triggered by coordinated transitions in gene expression during adipogenic differentiation might control intracellular variations on NAD^+^ levels and activity of NAD^+^-consuming enzymes. Indeed, we observed that NAMPT inhibition upregulated transcription of key lipid metabolism genes and adipocytic identity at terminal differentiation (Fig. 5, E–G), thus favoring lipid accumulation (Fig. 1 A). Indeed, other reports show that FK866-treated cells lose their adipogenic capacity (Okabe et al., 2020); however, these studies performed in mouse preadipocytes using higher concentrations of FK866, and these characteristics might underlie the reason for the divergent results. Yet, their results and ours align with the notion that the NAD^+^ salvage pathway fine-tunes adipogenesis in its late stages, which is also in line with its protective role in diet-induced obesity (Escalante-Covarrubias et al., 2022 *Preprint*; Stromsdorfer et al., 2016; Wang et al., 2017).

To gain insights into our observations in live, single cells at a submicrometric resolution, we developed a new method based on 2P-FLIM of intrinsic fluorophores that simultaneously provides directly quantitative metrics of adipogenic stem-cell differentiation and metabolic state of subcellular compartments. We measured a long lifetime of lipid droplets-associated fluorophores (Fig. 2, A and B), as previously shown (Alonzo et al., 2016; Datta et al., 2015). We used the lifetime contrast to select autofluorescence from lipids and from NADH within cells (Fig. S1, A and B). THG microscopy was performed to visualize lipid droplets (Chang et al., 2013; Débarre et al., 2006). Based on NADH intensity contrast, we also implemented an automated segmentation of mitochondria and cytoplasm/nuclear compartments that rely on their different concentrations of NADH (Fig. S1 B). With this method, we observed lipid accumulation and increased fraction of bound NADH during adipogenic differentiation in live single cells, reflecting the metabolic shift from glycolysis to OXPHOS metabolism during differentiation (Liu, 2018; Meleshina et al., 2016). We provided here the first description of intracellular NADH metabolic signature in different subcellular compartments and at distinct stages of adipogenic differentiation in human cells, since the subcellular characterization of NAD^+^/NADH metabolism was previously demonstrated during few hours after adipogenic induction from 3T3-L1 murine preadipocytes (Li et al., 2008; Ryu et al., 2018). Our results show that spatial subcellular compartmentalization of NADH metabolism is highly dependent on the differentiation stage and intimately linked with the transcriptional reprogramming allowing progressive lipid accumulation. Hereby, our observations are in agreement with the emerging view that temporal and spatial subcellular metabolic compartmentalization contributes to numerous biological roles and regulation of intracellular signaling and transcription (Aguilar-Arnal et al., 2016; Cambronne and Kraus, 2020) and that NAD^+^ compartmentalization regulates adipogenesis (Ryu et al., 2018).

Finally, the quantitative metrics based on fluorescence lifetime microscopy developed in this study serves as a label-free biomarker to simultaneously measure lipogenesis and metabolic shifts in single cell. Quantitative characterization of subcellular states from adipose tissues in health and disease using our two-photon microscopy-based method could provide means to uncover new roles of hMSC in obesity, thus paving the way for the development of MSC-based treatments.

## Materials and methods

### Isolation and characterization of bone marrow–MSCs (BM-MSCs)

BM-MSCs were obtained from healthy donors and have been previously described (Fajardo-Orduña et al., 2016), as follows: mononuclear cells from BM were obtained by density gradient centrifugation, and 2 × 10^5^ mononuclear cells/cm^2^ were seeded in low glucose DMEM (LG-DMEM, Gibco) with 10% FBS (Gibco), 4 mM L-glutamine and antibiotics. Cells were incubated at 37°C and 5% of CO_2_. At 80% of confluence, adherent cells were trypsinized and reseeded at a density of 0.2 × 10^4^ cells/cm^2^. Experiments were done at 3–5 passages. Cell surface markers’ expression of MSCs was determined by flow cytometry following our previously implemented and described protocols (Castro-Manrreza et al., 2014; Fajardo-Orduña et al., 2016), where MSCs were selected to express CD73, CD90, and CD105 markers, while being negative to the hematopoietic markers CD45, CD34, and CD14 (Fig. S5 A). Differentiation capacity was assessed using the StemPro Adipogenic and Osteogenic differentiation kits (A1007001, A1007201; Gibco), and the Chondrogenic Differentiation Medium (Cambrex Bio Science) supplemented with 10 ng/ml of TGFβ (100-21C; Peprotech), following manufacturer’s instructions. Adipogenic differentiation was evaluated by ORO (O0625; Sigma-Aldrich) staining after 16 d of induction, osteogenic differentiation was revealed by detecting alkaline phosphatase activity (B5655; Sigma-Aldrich) after 14 d of induction, and chondrogenic differentiation was indicated by the presence of mucopolysaccharides positive to alcian blue (ca. no. A5268; Sigma-Aldrich), in micromasses cross-sections, after 28 d of induction (Castro-Manrreza et al., 2014; Fajardo-Orduña et al., 2016). Representative images are shown in Fig. S5 B.

All protocols were compliant with the Declaration of Helsinki and approved by the Ethics Committee of Villacoapa Hospital, Mexican Institute for Social Security. Informed consent was given by the participants. Additionally, a BM-MSC line was purchased from ATCC.

### Cell culture and maintenance

hMSCs were maintained in LG-DMEM (cat. no. 31600-034; Gibco) supplemented with 10% FBS, 4 mM L-glutamine, 100 μg/ml of penicillin and streptomycin (Gibco) and incubated at 37°C and 5% of CO_2_. Adipogenic differentiation was induced in MSCs in growing phase (70–80% confluency), and drugs were present in the medium as indicated in the text and figures, whose concentrations had been previously standardized (Aguilar-Arnal et al., 2016). The medium and drugs were replaced every 4 d during the differentiation process. For live-cell imaging, BM-hMSCs (ATCC) were cultured on 3.5-cm glass-bottom petri dishes (MatTek) in 2.5 ml LG-DMEM per well without phenol red and 10% FBS and 100 UI/ml penicillin and 100 µg/ml streptomycin. Cells were plated at the initial density of 1.1 × 10^4^ cells/cm^2^ and allowed to attach overnight in a humidified cell culture incubator at 37 °C in 5% CO_2_ before proceeding with treatments. During the imaging experiments, we replaced the adipogenic medium with a basal medium without the phenol red before imaging. To determine the metabolic trajectory of NADH lifetime, MSCs were treated with rotenone 50 µM in DMSO (R8875; Sigma-Aldrich) and hydrogen peroxide (H_2_O_2_) 4 mM in DMSO (216763; Sigma-Aldrich), to block the respiratory chain through complex I and increase NAD^+^:NADH ratio through oxidative stress, respectively. All cultures tested negative for mycoplasma contamination.

hESC (H9 cell line) were purchased from WiCell Research Institute and were maintained in mTeSR Plus medium (100-0276; STEMCELL Technologies). For subculturing, hESCs were dissociated using Dispase (07923; STEMCELL Technologies) and then seeded onto plates coated with Geltrex LDEV-Free Reduced Growth Factor Basement Membrane Matrix (1:100; A1413202; Gibco) in medium containing 10 µM Y-27632 (1254; Tocris). The culture medium was replaced daily after the cells reached 30% confluency. Adipogenic differentiation was performed as described previously (Taura et al., 2009). Briefly, embryoid bodies were obtained from ESC colonies digested with dispase and plated onto ultra-low attachment 6-well plates (Corning), where they were allowed to aggregate in maintenance medium; DMEM/F-12 (Biological Industries) supplemented with 20% KnockOut serum replacement (10828028; Gibco), 1% non-essential amino acids, 1 mM L-Glutamine (01-340-1B and 03-020-1B; Biological Industries), 0.1 mM of β-Mercaptoethanol (Sigma-Aldrich), and 1× penicillin-streptomycin (15140122; Gibco). Retinoic acid (R2625; Sigma-Aldrich) was added to a concentration of 100 nM from days 2–5. After 12 d, embryoid bodies were transferred to 6-well or 12-well plates coated with Geltrex 1:100, and adipogenic differentiation was induced for 10 d using MesenCult Adipogenic Differentiation Kit medium (StemCells Technologies), changing the medium every 2 d.

### Antibodies and reagents

The antibodies used in this study are as follows: anti-SIRT1, cat. no. 07-131, Millipore; anti-PPARγ, cat. no. 81B8, Cell Signaling; anti-GAPDH-HRP conjugated, cat. no. GTX627408-01, GeneTex; Goat anti-Rabbit Alexa Fluor 594 conjugated, cat. no. R37117, Invitrogen; anti rabbit IgG-HRP conjugated, cat. no. 65-6120, Invitrogen. All purchased antibodies were validated for mammalian studies (as shown on the manufacturers’ websites). EX527 (E7034), FK866 (F8557), and NAD^+^ (N8535) were purchased from Sigma-Aldrich. β-NMN was purchased from Santa Cruz Biotechnology (sc-212376).

### ORO staining

Cells grown on slides were briefly washed with PBS and fixed for 45 min with 4% fresh paraformaldehyde. Preparation of ORO (cat. no.O1391; Sigma-Aldrich) working solution and staining of slides were performed as described (Mehlem et al., 2013). ORO was applied on the slides for 5 min at RT. Slides were washed twice during 10 min in water and mounted in vectashield mounting media (cat. no. H-1000; Vector Labs). The images were captured with the camera Axiocam EEc 5 s coupled to a ZEISS Primovert microscope, using a 20× magnification at RT. Lipid droplets were quantified using the ImageJ software, by converting RGB to 8-bit grayscale images, and then using the “analyze particles” plug-in (Collins, 2007). Four frames per slide were used for image analyses and quantification (*n* = 3 biological replicates with 4–7 technical replicates).

### Quantitative real-time PCR

Total RNA was extracted from MSCs using TRIzol Reagent (cat. no. 15596018; Invitrogen) following the manufacturer’s instructions. cDNA was obtained by retrotranscription of 1 μg of total mRNA with iScript cDNA synthesis kit (Bio-Rad) according to the manufacturer’s instructions. Real-time RT-PCR was done with the real-time CFX96 detection system (Bio-Rad). For a 10 μl PCR reaction, 25 ng of cDNA template was mixed with the primers to final concentrations of 200 nM and mixed with 5 μl of iTaqTM Universal SYBR Green Supermix kit (Bio-Rad). The reactions were done in triplicates with the following conditions: 30 s at 95°C, followed by 45 cycles of 30 s at 95°C and 30 s at 60°C. Expression levels were calculated using the ddCt method. The quantitative PCR (qPCR) primers are described in Table S5.

### Immunofluorescences

MSCs or H9 cells were seeded on Lab-Tek Chamber slides (Thermo Fisher Scientific) at 9 × 10^3^ cells/cm^2^. After 36 h of incubation, cells were washed with PBS (Gibco), fixed with 1% of paraformaldehyde at 37°C for 10 min, washed twice with PBS and permeabilized with PBS 0.1% triton during 15 min. The slides were then washed with PBS and incubated with blocking buffer (PBS, 0.1% Tween 20, 2% BSA) for 1 h. Incubation with anti-SIRT1 (1:500) was performed over night at 4°C, and the secondary antibody (1:2,000) was incubated during 1 h at RT. 1:5,000 dilution of Hoechst 33342 was used for nuclear counterstain (H1399; Invitrogen) by incubating during 10 min at RT. Coverslips were mounted using VECTASHIELD antifade mounting medium (H1000; Vector labs) and sealed with nail polish. Fluorescence images were acquired by an Olympus DP70 Digital Camera in an Olympus BX51 fluorescence microscope. Hoescht stain was acquired at a 1/300 s exposure, while SIRT1 intensity was acquired at 1/200 s, at room temperature. Densitometry was performed using ImageJ on three cells at four different fields from two biological replicates.

### Western blotting

MSCs were harvested from confluent 6-well dishes, washed with PBS, and lysed with radioimmunoprecipiation buffer supplemented with HDAC inhibitors (50 mM Tris, pH 8, 150 mM NaCl, 1% 5 mM EDTA, pH 8, 1% NP-40, 0.5% Na deoxycholate, and 0.1% SDS; all from Sigma-Aldrich). Cells were left on ice during 20 min and centrifuged at 14,000 rpm during 15 min at 4°C. The protein extracts in the supernatants were snap-frozen and stored at −80°C. Protein quantification was done using the Bradford colorimetric assay (B6916; Sigma-Aldrich). 20 µg of proteins were separated run on a 10% SDS-PAGE gel, at 100 V for 2 h using a mini-PROTEAN system (BIO-RAD) and transferred to nitrocellulose membrane (Millipore) at 40 mV overnight at 4°C. A 1:1,500 dilution of anti-PPARγ in blocking buffer (Tris-buffered saline plus Tween-20 and 5% nonfat milk) was used to detect the protein. Antibodies to GAPDH protein at 1:20,000 dilution in PBST (137 mM NaCl, 2.7 mM KCl, 10 mM Na_2_HPO_4_, 1.8 mM KH_2_PO_4_, and 1% Tween 20) were used as a loading control. Proteins were revealed using chemiluminescent detection (Immobilon Western, WBKLS0100; Millipore) and visualized using a Kodak GEL Logic 1,500 Imaging System with Transilluminator.

### Generation of SIRT1 knockdown in H9 cells using CRISPR-Cas9

The plasmid pSpCas9(BB)-2A-Puro (PX459) V2.0 previously synthesized (Ran et al., 2013) was purchased from Addgene (plasmid # 62988; Addgene). The shot-guide RNAs (sgRNAs) were designed using Benchling to edit the *SIRT1 locus* at the Exon 4 (SIRTE4 sgRNA38 Fw and Rv, Table S5). The oligos were phosphorylated using T4-Polynucleotide Kinase (EK0031; Invitrogen), and annealed in a thermal cycler at 37°C 30 min, 95°C 5 min, and 1°C was decreased each 30 seg, to 25°C. The PX459 plasmid was digested with *BbsI* (R0539; NEB), and the double-stranded sgRNA was inserted with T4 ligase (EL0011; Invitrogen). The final construct was transformed in TOP10 and isolated using a Plasmid Midi Preparation Kit (Promega). 2 μg of this plasmid were electroporated in 2 × 10^5^ H9 cells using the neon electroporator (Thermo Fisher Scientific) with 10 μl tips. The electroporator was set to 1,050 V, 30 ms, and 2 pulses. Additionally, a non-related sgRNA designed against mCherry sequence was used in parallel, as a control (mCherry sgRNA Fw and Rv, Table S5). Electroporated cells were grown in 12-well plates for 24 h, and then the medium was replaced with fresh medium containing 0.5 μg/ml of puromycin which was maintained for 48 h. After this selection, cells were recovered and a limiting dilution of the pool of resistant cells was performed in 96-well plates. Individual clones, after their expansion, were validated by genotypification using the primers SIRTE4_Fw y Rv (Table S5). Sanger sequencing, RT-qPCR, and Western blotting were used for further validation. The most probable off-targets were identified using Benchling as non-coding sequences, and PCR genotypification followed by sequencing was used to discard off targets from the CRISPR system (OFF Tgt 38 sgRNA primers in Table S5).

### Extracellular flux analysis

OCR and ECAR were measured using the Seahorse XFe96 Analyzer (Agilent), using The Cell Mito Stress Test Kit (cat. no. 103015-100; Agilent). MSCs were seeded at a density of 10,000 cells/well in a XF96 cell culture 96-well microplate (101085-004; Agilent) precoated with 10 µg/ml of fibronectin (F1141; Sigma-Aldrich). Adipogenic induction and treatments were initiated after 2 d of seeding. 1 h prior the Seahorse analysis, MSCs cultures were washed with 200 µl/well of XF assay media supplemented with 10 mM glucose, 2 mM glutamine, and 1 mM pyruvate. Then, 180 µl/well of this medium was added, then the plate was equilibrated for 30 min at 37°C in a CO_2_-free incubator before being transferred to the Seahorse XFe96 analyzer. Measurement of OCR and ECAR was done at baseline and following sequential injections of (i) 2 μM oligomycin, an ATP synthase inhibitor, (ii) 0.5 μM carbonyl cyanide-4-(trifluoromethoxy) phenylhydrazone (FCCP), a protonophoric uncoupler, and (iii) 0.5 μM of rotenone, an inhibitor of complex I of the electron transport chain. Briefly, oligomycin inhibits mitochondrial ATP synthase, and the resulting drop in OCR and rise in ECAR are attributed to ATP-linked OCR and the compensation of glycolysis for the loss of mitochondrial ATP production. FCCP uncouples the mitochondrial proton gradient and oxygen consumption from ATP synthase, hereby driving maximal OCR. Rotenone inhibits complex I of the electron transport chain; hence, it hinders mitochondrial oxygen consumption. Therefore, the residual OCR is regarded as non-mitochondrial.

### Total NAD^+^ and NADH measurements

Total NAD^+^ and NADH were determined from 2 × 10^5^ hMSCs using the NAD/NADH colorimetric assay (Cat. No. 8368; Sciencell Research), following manufacturer’s instructions. Absorbance at 490 nm was measured using a BioTek Synergy plate reader. Samples were analyzed per triplicates.

### Expression profiling (RNA-seq) and analysis

Total RNA from MSCs was extracted using Quick RNA MiniPrep Kit (Zymo Research) following the manufacturer’s instructions. RNA samples with RNA integrity number >7.0 were sent for library preparation and sequencing to Novogene Corporation Inc. Briefly, mRNA was isolated using oligo(dT) beads and randomly fragmented by adding fragmentation buffer, followed by cDNA synthesis primed with random hexamers. Next, a second-strand synthesis buffer (Illumina), dNTPs, RNase H, and DNA polymerase I were added for second-strand synthesis. After end repair, barcode ligation, and sequencing adaptor ligation, the double-stranded cDNA library was completed with size selection to 250–300 bp and PCR enrichment. Sequencing was performed on an Illumina NovaSeq 6000 Sequencing System with paired-end 150 bp reads, at 9 G raw data/sample. Total and mapped reads per sample are shown in Table S1.

### RNA-seq data processing

Human Reference genome and gene model annotation files were downloaded from the genome website browser (NCBI/UCSC/Ensembl). Indexes of the reference genome were built using STAR and the paired-end clean reads were aligned to the *Homo sapiens* assembly GRCh38/hg38, with the STAR aligner v2.5 (Dobin et al., 2012). STAR uses the method of maximal mappable prefix, which can generate a precise mapping result for junction reads. HTSeq v0.6.1 was used to count the read numbers mapped of each gene. Afterwards, the Fragments Per Kilobase of transcript per Million mapped fragments of each gene was calculated based on the length of the gene and reads count mapped to it (Mortazavi et al., 2008). Differential expression analysis between conditions (three biological replicates per condition) was performed with the DESeq2 R package (2_1.6.3), which uses a model based on the negative binomial distribution (Love et al., 2014). The resulting P values were adjusted using Benjamini and Hochberg’s approach for controlling the false discovery rate (FDR). Genes with an adjusted P value <0.05 were assigned as DE. The Venn diagrams were prepared using the function vennDiagram in R based on the gene list for different groups, or with Venny V 2.1 (https://bioinfogp.cnb.csic.es/tools/venny/). DE genes were subjected to functional analyses using the “Compute Overlaps” tool to explore overlap with the canonical pathways) and the GO biological process gene sets at the MSigDB (molecular signature database). The tool is available at https://www.gsea-msigdb.org/gsea/msigdb/annotate.jsp and estimates statistical significance by calculating the FDR q-value. This is the FDR analog of the hypergeometric P value after correction for multiple hypotheses testing according to Benjamini and Hochberg. GSEA was performed using GSEA v. 4.0.3. (Subramanian et al., 2005) to determine the enrichment score within the Hallmark gene set collection in MSigDB v.7.0 (Liberzon et al., 2015), selecting the Signal2Noise as the metric for ranking genes. The findMotifs.pl program in the HOMER software (Heinz et al., 2010) was used for motif discovery and enrichment, searching within the genomic regions encompassing 300 Kb upstream and 50 Kb downstream the TSS, and selecting 6–8 bp for motif length. Motif enrichment is calculated by findMotifs.pl using the cumulative hypergeometric distribution.

All raw and processed RNA-seq data are publicly available at Gene Expression Omnibus under the accession number GSE178615.

### FLIM and THG

Live imaging was performed on MSCs in basal medium without phenol red, at 37°C and 5% CO_2_, using a laser scanning microscope (TriMScope, Lavision Biotec). A simplified scheme of the multiphoton microscope is shown in Fig. S5 C. The excitation is provided by a dual-output femtosecond laser (Insight DS++, Spectra-Physics) with a first beam turnable from 680 to 1,300 nm (120 fs pulses, 80 MHz) and a second, fixed wavelength beam at 1,040 nm (200 fs pulses). A water Immersion objective (25×, NA = 1.05, XLPLN-MP; Olympus) is used to focus the laser on the sample and collect fluoresce signal. Fluorescence signal is epi-detected by a hybrid photomultiplier tube (R10467U; Hamamatsu), whereas THG signal is forward-detected by a photomultiplier (H6780-01; Hamamatsu). To perform FLIM of NADH, 760-nm wavelength excitation was used with a typical power of 12 mW (Fig. S5 D). A band-pass filter was used in front of the detector to collect NADH autofluorescence (FF01–460/80; Semrock). Time-correlated single photon counting electronics (Lavision Biotec) with 5.5 ns dead time and 27 ps time bins were used to measure the arrival time of the fluorescence photons with respect to the laser pulse and perform FLIM imaging. The laser trigger reference was taken from the fixed wavelength beam using a photodiode (PDA10CF-EC; Thorlab). Calibration of the FLIM system was performed by measuring the lifetime of fluorescein at pH = 9 with a single exponential of 4 ns (Fig. S5 E). We measured the lifetime of free NADH in solution (n. N8129; Sigma-Aldrich) to calculate the fraction of bound NADH (Fig. S4 B). We typically collected 500 photons for FLIM images of live cells with a pixel dwell time of 240 μs/pixel and a total acquisition time on the order of 1 min. THG was performed using a wavelength of 1,100 nm with a typical power of 12 mW, and the signal was collected with a band-pass filter (FF01–377/50; Semrock; Fig. S5 D). We typically collected 800 photons for THG images with a pixel dwell time of 53 μs/pixel and a total acquisition time of the order of 1 min. Raw images can be accessed at https://zenodo.org/record/6773422#.YtrR2oRByUk.

### Analysis of the FLIM images

Intensity images were analyzed with Fiji-ImageJ (National Institutes of Health). All FLIM data were processed and analyzed with SimFCS (developed by the laboratory for Fluorescence Dynamics; https://www.lfd.uci.edu/globals/) and with a MATLAB (MathWorks) custom-written software. FLIM data were transformed using Fast Fourier Transform (FFT) and plotted in the phasor plot following previously described methods (Ranjit et al., 2018; Stringari et al., 2011), as follows: The raw FLIM data in the form of time domain displays a multi-exponential decay (Fig. S1 A). Based on FT, the multi-exponential fluorescence intensity decay in every pixel of the image is transformed in phasor plot by these expressions:

$$g_{i,j}\left(\omega \right)=\frac{\int_{0}^{\infty }I_{i,j}\left(t\right)*\cos\left(\omega t\right)dt}{\int_{0}^{\infty }I_{i,j}\left(t\right)dt}\text{.}$$ (1)

$$s_{i,j}\left(\omega \right)=\frac{\int_{0}^{\infty }I_{i,j}\left(t\right)*\sin\left(\omega t\right)dt}{\int_{0}^{\infty }I_{i,j}\left(t\right)dt}\text{,}$$ (2)

where *i* and *j* indicate the order pixel of the image and ω is the frequency. ω is calculated through the laser repetition rate ω = 2πf. Then in the frequency domain, the FLIM data are presented as:

$$g_{i},\left(\omega \right)=m_{i,j}{\cos\varphi }_{i,j}\text{.}$$ (3)

$$s_{i},\left(\omega \right)=m_{i,j}\sin\varphi_{i,j}\text{,}$$ (4)

where m_i_,_j_ and φ_i,j_ present the phase-shift and phase demodulation between the excitation and emission signal. The lifetime in terms of phase and modulation then can be expressed by these following formulas:

$$\tau_{\varphi }=\frac{1}{\omega }\tan\left(\varphi \right)\text{.}$$ (5)

$$\tau_{m}=\frac{1}{\omega }\sqrt{\frac{1}{m^{2}}-1}\text{.}$$ (6)

In order to evaluate the lifetime shift between days of experiment, we did use a solution of fluorescein at pH 9 to have single lifetime at 4.04 ns as a reference (Fig. S5 F). Some endogenous biomarkers in solution were tested on the FLIM system. The coordinates *g* and *s* in the phasor plot were calculated from the fluorescence intensity decay of each pixel of the image using the transformations defined in Eqs. 1 and 2. We applied an intensity threshold to eliminate the background of the cellular medium and a median filter on the *g* and *s* images to reduce the variance of the phasor location without decreasing the spatial resolution of the image (Stringari et al., 2017). For every pixel of the image, we calculated the value of τ_*φ*_ (Eq. 5) and τ_*m*_ (Eq. 6) starting from the g and s images (Fig. S1 A). Fluorescein, free NADH, and free FAD have single lifetime which locate on the universal circle of the phasor plot. On the other hand, bound NADH presents a multi-exponential lifetime that locates inside the universal circle. To quantify the ratio NADH/NAD^+^, we used fraction of bound NADH which is graphically calculated as the distance from the experimental point to the point of free NADH on the phasor circle (Figs. S1 A and S5 F):

$$fB_{NADH}=\sqrt{{\left(g_{\exp}-g_{fNADH}\right)}^{2}+{\left(s_{\exp}-s_{fNADH}\right)}^{2}}\text{.}$$ (7)

Raw images can be accessed at https://zenodo.org/record/6773422#.YtrR2oRByUk.

### Subcellular segmentation of lipid droplets, cell NADH, mitochondria, and nucleus and cytoplasm

Image processing and segmentation were performed by MATLAB custom-written software. The principles of the segmentation are illustrated in Fig. S1 B. A threshold (2.87 ns) was applied into the τ_m_ lifetime image to automatically separate lipid droplets and NADH of the cell. Pixels with longer lifetime were assigned to a lipid mask while pixels with shorter lifetime were highlighted to the NADH cell mask. The τ_m_ threshold was determined empirically to match the lipid mask border with the THG signal of the lipid droplet (Fig. 2 A). Then, the mask of NADH cells was used to calculate fB_NADH of the same region of interest (ROI) or cell. For quantification, we used the average values of fB_NADH and lipid ratio. To quantify the lipid droplets in ROIs or in single cells, we calculated the ratio between the number of pixels of the lipid mask and the total number pixels of the cell; single-cell analysis was performed manually. We performed subcellular segmentation of mitochondria and nucleus plus cytoplasm applying a threshold (150 photons) to the intensity image multiplied with the NADH mask (Fig. S1 B). The threshold was determined based on the different NAD^+^/NADH ratios in mitochondria (∼10) and cytoplasm and nucleus (∼50–1,000; Stein and Imai, 2012). Pixels with higher number of photons were assigned to mitochondria while pixels with lower number of photons are assigned to nucleus and cytoplasm. The masks of mitochondria and nucleus and cytoplasm were applied to the map of fraction of bound NADH, and the average fraction of bound NADH was calculated in different cellular compartments for statistical analysis.

### Statistics and images analyses

Data are shown as mean with SEM. Statistical analyses were performed using GraphPad Prism 8.2. The statistical tests were performed as indicated in the figure legends, mostly consisting of two-way ANOVA followed by Tukey’s multiple comparisons test, or Kruskal–Wallis H-test. Statistical significance was considered when the P value was <0.05. Unless stated otherwise, data distribution was assumed to be normal, but this was not formally tested. When possible, experimental evaluation was performed blind to the experimental conditions (i.e., specifically for Western blot quantification, image processing, and subsequent quantifications). Western blot analyses and image processing for overlay in different channels from immunofluorescences were performed with ImageJ software. Figures were arranged using Adobe Illustrator.

### Online supplementary material

Fig. S1 (in support of Fig. 1) shows the image processing workflow for FLIM and lipids, and NADH segmentation, carefully described also in the Materials and methods section. A control Western blot for PPARγ expression in MSCs is also shown. Fig. S2 (in support of Fig. 3) shows complementary RNA-seq data analyses from MSCs. Fig. S3 (in support of Figs. 4, 5, and 6) shows GO analyses from EX527-treated MSCs, and also the images from SIRT1 immunofluorescences showing changes in SIRT1 expression and subcellular location during adipogenesis from untreated or treated MSCs. Proton leak and non-mitochondrial respiration was quantified from the seahorse data presented in Fig. 6, while NAD^+^/NADH quantifications are also shown. Fig. S4 (in support of Fig. 7) describes the identification of the metabolic trajectory between free NADH and bound NADH, and shows quantifications for the fraction of bound NADH in mitochondria compared to the nucleus and cytoplasm in MSCs. Fig. S5 supports the Materials and methods section, showing characterization of bone marrow–derived human MSCs, and the microscopy set up and procedures for FLIM analyses using the phasor plot. Table S1 is an Excel file describing the quality of the RNA-seq data, regarding sequencing depth and the mapping rate. Table S2 is an Excel file supporting Fig. 3 and contains the list of DE genes from NAD^+^-treated cells and their functional analyses. Table S3 is an Excel file supporting Fig. 4 and shows the list of DE genes from EX527-treated cells and their functional analyses. Table S4 is an Excel file supporting Fig. 5 and shows the list of DE genes from FK866-treated cells and their functional analyses. Table S5 contains the list of primers used in this study.

## Supplementary Material

Table S1 is a summary of mapping results.

Table S2 lists DE genes from NAD^+^-treated cells genes and their functional analyses.

Table S3 lists DE genes in from EX527-treated cells at terminal differentiation (day 16) and their functional analyses.

Table S4 lists DE genes in from FK866-treated cells at terminal differentiation (day 16) and their functional analyses.

Table S5 lists primers used in this study.

SourceData F1 contains original blots for Fig. 1.

SourceData F2 contains original blots for Fig. 2.

SourceData FS1 contains original blots for Fig. S1.

## Data Availability

RNA-seq data is in Gene Expression Omnibus accession no. GSE178615 (https://www.ncbi.nlm.nih.gov/geo/query/acc.cgi?acc=GSE178615). Image analysis scripts are on GitHub (https://github.com/laguilar7/Axolot). Raw images are at https://zenodo.org/record/6773422#.YtrR2oRByUk. Any other relevant data to this manuscript are available from the authors.
