# Supplementary figures and images for: Coordinated metabolic transitions and gene expression by NAD+ during adipogenesis
Source: J Cell Biol. 2022 Oct 5;221(12):e202111137. doi: 10.1083/jcb.202111137 (PMC9538974; doi:10.1083/jcb.202111137)

1F

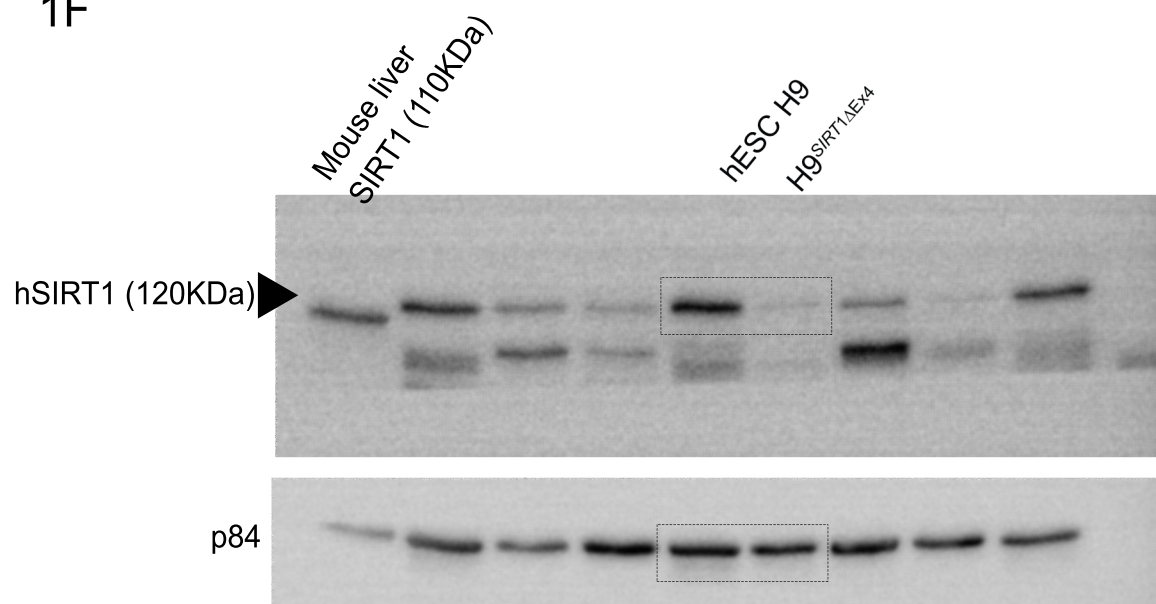

Supplement: SourceData F1 — contains original blots for Fig. 1. [file JCB_202111137_SourceDataF1.pdf]

2D

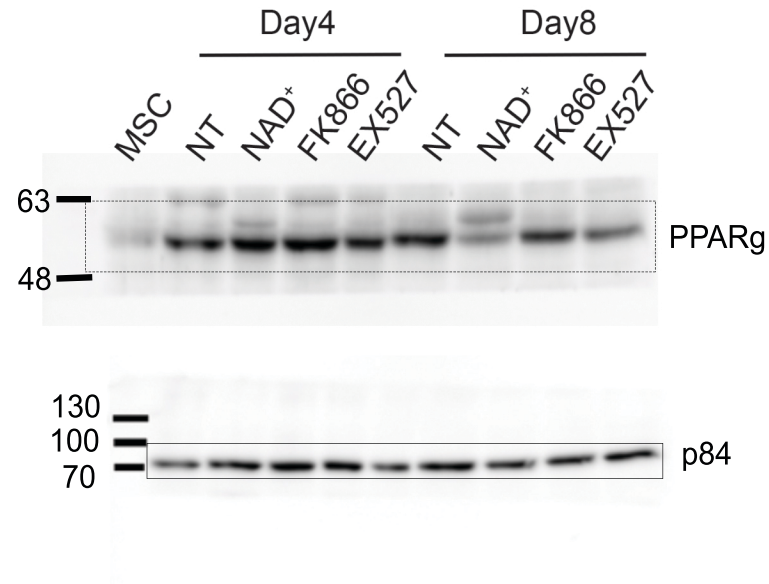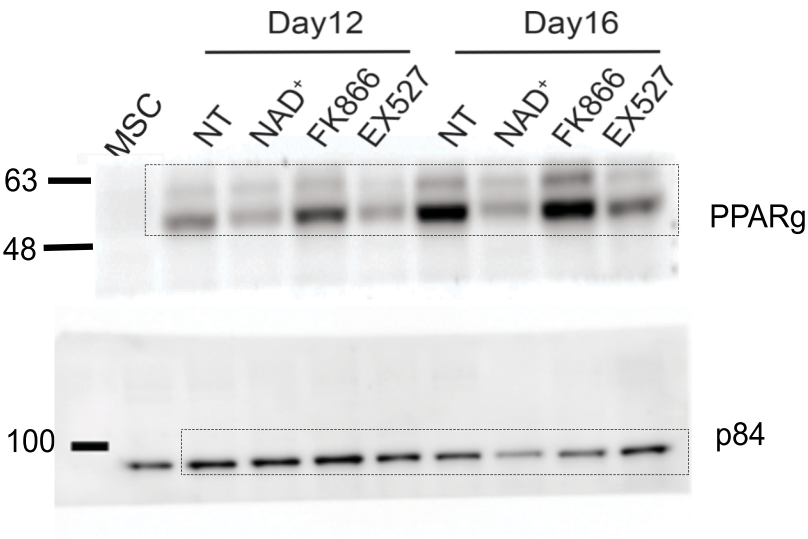

2F

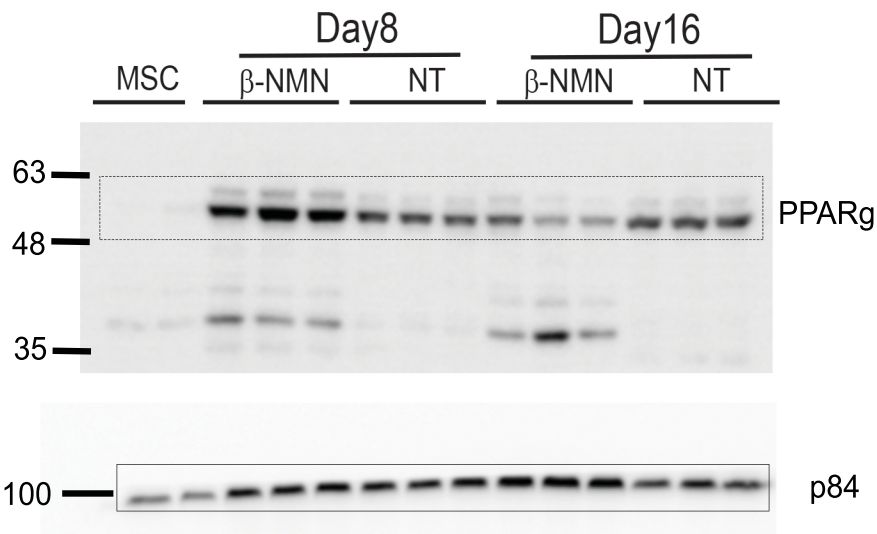

Supplement: SourceData F2 — contains original blots for Fig. 2. [file JCB_202111137_SourceDataF2.pdf]

S1C

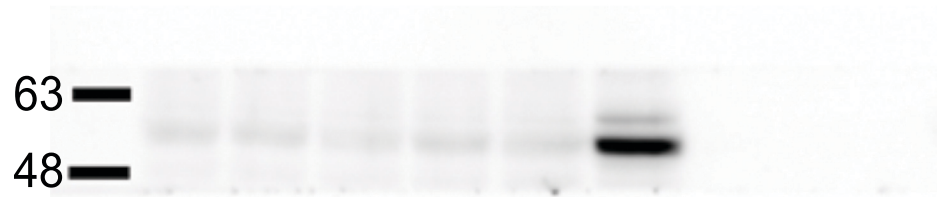

PPAR $\gamma$

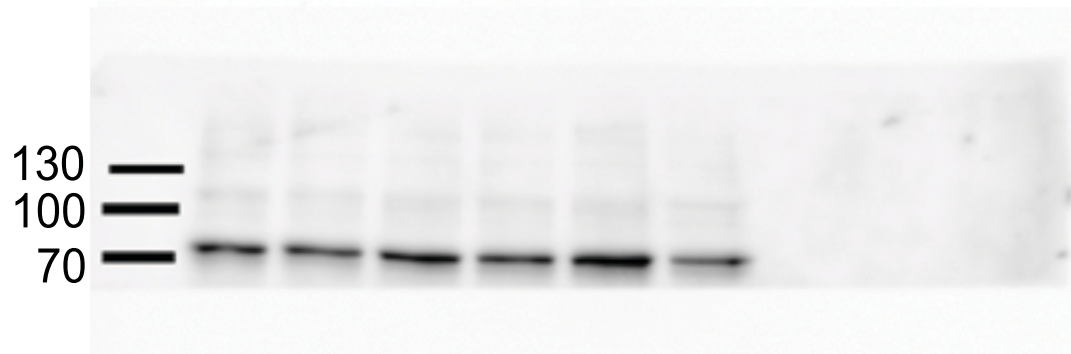

p84

Supplement: SourceData FS1 — contains original blots for Fig. S1. [file JCB_202111137_SourceDataFS1.pdf]
